# Approaching cellular resolution and reliable identification in mass spectrometry imaging of tryptic peptides

**DOI:** 10.1007/s00216-018-1199-z

**Published:** 2018-08-01

**Authors:** Katharina Huber, Pegah Khamehgir-Silz, Thorsten Schramm, Vladimir Gorshkov, Bernhard Spengler, Andreas Römpp

**Affiliations:** 1 Institute of Inorganic and Analytical Chemistry, Justus Liebig University, Heinrich-Buff-Ring 17, 35392 Giessen, Germany; 2 Bioanalytical Sciences and Food Analysis, University of Bayreuth, Universitaetsstrasse 30, 95440 Bayreuth, Germany

**Keywords:** Mass spectrometry imaging, On-tissue digestion, High mass resolution, Data format imzML, Protein identification

## Abstract

On-tissue digestion has become the preferred method to identify proteins in mass spectrometry (MS) imaging. In this study, we report advances in data acquisition and protein identification for MS imaging after on-tissue digestion. Tryptic peptides in a coronal mouse brain section were measured at 50 μm pixel size and revealed detailed histological structures, e.g., the ependyma (consisting of one to two cell layers), which was confirmed by H&E staining. This demonstrates that MS imaging of tryptic peptides at or close to cellular resolution is within reach. We also describe a detailed identification workflow which resulted in the identification of 99 proteins (with 435 corresponding peptides), based on comparison with LC-MS/MS data and in silico digest. These results were obtained with stringent parameters, including high mass accuracy in imaging mode (RSME < 3 ppm) and at least two unique peptides per protein showing consistent spatial distribution. We identified almost 50% of proteins with at least four corresponding peptides. As there is no agreed approach for identification of proteins after on-tissue digestion yet, we discuss our workflow in detail and make the corresponding mass spectral data available as “open data” via ProteomeXchange (identifier PXD003172). With this, we would like to contribute to a more effective discussion and the development of new approaches for tryptic peptide identification in MS imaging. From an experimental point of view, we demonstrate the improvement due to the combination of high spatial resolution and high mass resolution/mass accuracy on a measurement at 25 μm pixel size in mouse cerebellum tissue. A whole body section of a mouse pub imaged at 50 μm pixel size (40 GB, 230,000 spectra) demonstrates the stability of our protocol. For this data set, we developed a workflow that is based on conversion to the common data format imzML and sequential application of freely available software tools. In combination, the presented results for spatial resolution, protein identification, and data processing constitute significant improvements for the field of on-tissue digestion.

**Electronic supplementary material:**

The online version of this article (10.1007/s00216-018-1199-z) contains supplementary material, which is available to authorized users.

**Graphical abstract:**
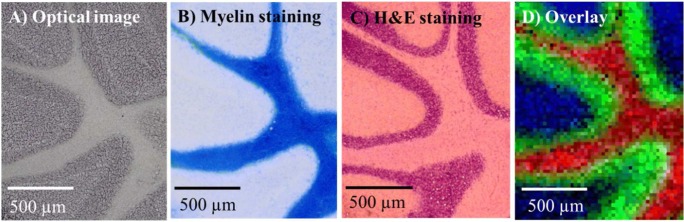
MS imaging of coronal mouse brain cerebellum with a pixel size of 25 μm: **A** Optical image, **B** myelin staining, **C** H&E staining, and **D** MS image overlay (RGB) of tryptic peptides *m*/*z* = 726.4045 ± 0.005, HGFLPR + H^+^ (red), *m*/*z* = 536.3173 ± 0.005, AKPAK + Na^+^ (green), and *m*/*z* = 994.5436 ± 0.005, WRQLIEK + Na^+^ (blue)

## Introduction

Mass spectrometry (MS) imaging provides information about the spatial distribution of an analyte in a (biological) sample. In matrix-assisted laser desorption/ionization (MALDI) MS, a focused laser beam is used to generate ions which are analyzed in the mass spectrometer. A full mass spectrum is generated for each position sequentially. The intensity distribution of each mass peak (corresponding to a certain compound) can be displayed as an “image.” Individual MS images can be generated for each signal in the mass spectrum. Therefore, MS imaging is an “untargeted” and multiplexed method giving it an advantage compared with other molecular imaging techniques, e.g., histochemical staining which depends on the availability of suitable antibodies.

MALDI imaging has evolved into a widely used analytical technique in recent years [1–3]. Numerous applications have been published including the imaging of lipids [4], peptides [5, 6], proteins [7], and drug compounds [8]. A general overview of state of the art of MS imaging methodology with a focus on spatial resolution and reliable molecular identification can be found in a recent review article [9].

Although significant progress has been made in MS imaging in recent years, a number of challenges still remain. A question that often arises in discussions with biologists and medical researchers is regarding the identification (and ideally quantification) of proteins in individual cells. This kind of information is not accessible with any technique available (for a larger number of proteins in one experiment). Current MS imaging techniques are limited in spatial resolution and/or detection specificity.

Proteins have been extensively studied in biomedical research. The analysis of their spatial distribution can provide additional information about their role in physiological and pathological processes [10]. Numerous studies have been published on MS imaging of proteins, examples include references [11–13]. Lipids are usually removed by a series of washing steps prior to analysis [14]. Spatial resolution of reported protein imaging analyses is often in the range of 100 to 250 μm. Notable exceptions are studies at 10 μm pixel size [15] and 2.5 μm pixel size [16] with time-of-flight (TOF) mass analyzers.

Nevertheless, the analysis of proteins by MS imaging is still a challenging task [10, 17]. A major drawback of many studies is also that protein signals are often not identified but merely reported as *m/z* values. This limitation reduces the relevance of results reported for many biomedical applications significantly. Identification of intact proteins from tissue is difficult due to the limited mass resolution of time-of-flight mass spectrometers (TOF-MS), which is insufficient to resolve the complexity of these samples. A promising approach is the detection of intact proteins by Fourier transform ion cyclotron resonance mass spectrometry (FTICR-MS) [18, 19]. A general limitation of direct analysis of intact proteins under imaging conditions is the limited mass range (typically up to 25 kDa), i.e., the majority of proteins cannot be detected. And even in this mass range, only the most abundant proteins (typically100 to 200 mass peaks, compared with thousands of proteins which are present in the tissue) can be detected. A possible approach is to tentatively identify intact proteins by additional liquid chromatography (LC)-MS/MS measurements (of an adjacent tissue section) and to validate them by specific antibodies [20, 21].

An alternative approach that is increasingly used is to digest proteins by applying enzyme solution directly onto the tissue section. Trypsin is typically used as the enzyme, and the distribution of the resulting tryptic peptides is imaged [22, 23]. Peptides are easier to detect and identify than the intact proteins due to their lower molecular weight. This technique has been used for the analysis of tumor samples and several other tissue types [24–26]. Another advantage of enzymatic on-tissue digestion is that formalin-fixed paraffin-embedded (FFPE) tissue can be analyzed and thus gives access to a vast number of clinically relevant samples [23, 27–29].

In early studies, trypsin was deposited on discrete positions by a spotting device [22, 30]. Most current studies use homogeneous application of trypsin by spraying. Application devices include pneumatic sprayers [25], “vibrational vaporization” [31, 32] or homebuilt sprayers [33]. In any case, it is crucial that experimental parameters need to be carefully controlled and optimized as trypsin application is the most critical factor for the molecular information content and spatial resolution of the resulting MS images. A number of approaches have been proposed in recent years to optimize the on-tissue digestion workflow for MS imaging. The influence of different sample preparation steps have been systematically studied by Diehl et al. [29]. Dekker et al. have compared on-tissue digestion result in two different laboratories [34]. The number of peptides detected was increased in a study with different enzymes for on-tissue digestion [35]. Djidja et al. have shown an increased yield of tryptic peptides by addition of a detergent in the digestion workflow [36]. Humidity and temperature during the digestion process can be controlled with specifically designed reaction chambers [37]. The ionization efficiency of tryptic peptides can be increased by an additional derivatization step [38].

Most results for tryptic peptides are still limited to highly abundant proteins. Typically, no more than a few tens of peptides are identified by MS/MS measurements directly on tissue (while many more potential peptide peaks are detected). This is due to the limited fragmentation efficiency of singly charged ions, which are predominately produced in MALDI, and to ion suppression effects in the complex matrix of biological tissue. An approach to resolve the high complexity of tissue sections is the use of ion mobility as an additional separation step, which has also been applied for MS imaging of tryptic peptides [36, 39, 40].

Alternatively, the number of identified peptides can be increased by a combination of MS imaging with LC coupled to MS (LC-MS). The chromatographic separation reduces ion suppression effects and peptide ions produced in electrospray are typically multiply charged and, thus, result in richer fragment ion spectra. In this approach, one tissue section is analyzed by MS imaging, while an adjacent section is homogenized and analyzed by LC-MS/MS. This approach has been applied in several studies [20, 27, 30, 41]. Linking the two MS modalities is, however, challenging due to the high complexity of the biological samples analyzed (mainly mammalian tissue sections), which can lead to false-positive assignments if the mass accuracy is not high enough as demonstrated by Schober et al. [42] or if there are unaccounted for sources of complexity in the search parameters (e.g., post-translational modifications). Many data of tryptic peptides are acquired on axial MALDI-TOF instruments. For tissue imaging conditions, these mass spectrometers provide mass accuracies of around 100 ppm for carefully optimized measurements, which is usually insufficient for direct identification of tryptic peptides. One study reported mass accuracies of 20 to 30 ppm for peptides of selected proteins which were acquired at 250 μm pixel size [22]. A strategy to recalibrate data from axial MALDI-TOF instruments was published recently [32]. The authors reported an improvement by a factor of up to 10 for mass accuracy. Values for mass deviation after recalibration were between 1 and 60 ppm. Measurements with orthogonal TOF systems, which are less prone to mass shifts due to tissue topology, usually result in mass accuracies of about 30 ppm (at 200 μm pixel size) [25].

The first tissue imaging measurement with accurate mass for tryptic peptides (root mean square error of less than 3 ppm for all detected peptides) was reported by Schober et al. [42] using an Orbitrap mass spectrometer. This data (acquired at 100 μm pixel size) provided much more reliable results for identification of peptides directly from tissue. The study also demonstrated that a combination of MS imaging with a dedicated LC-MS/MS protocol can increase the number of identified peptides significantly. A similar approach was used by Quanico et al. [43] to acquire accurate mass data at 300 μm pixel size. An interesting addition in this study was the use of liquid extraction surface analysis (LESA) instead of the analysis of homogenized tissue as a complementary method for identification.

The detection of single cells requires a spatial resolution in the range of 10 to 20 μm. The pixel size for MS imaging of tryptic peptides was typically in the range of 100 to 200 μm. It should also be noted that a small pixel size alone does not necessarily result in high spatial resolution. Sample preparation can affect the distribution of the compounds analyzed (tryptic peptides in this case) or low signal intensities can lead to “noisy” MS images with very limited spatial information. The quality of spatial information should be evaluated based on the comparison with optical techniques, typically after histological staining of the tissue measured (or an adjacent section). In a previous study, we reported the first data with a pixel size of less than 100 μm for tryptic peptide imaging [33]. The MS images were acquired with 50 μm pixel size and a mass accuracy of better than 3 ppm. The MS images were generated with a bin width of Δ*m/z* = ± 0.01. A study reported MS images acquired at 30 μm pixel size [44]. Mass accuracy was not reported for this data, but another measurement in the same study acquired at 150 μm pixel size resulted in mass accuracies of up to 50 ppm and the images were generated with a bin width of Δ*m/z* = ± 0.2. A combination of a pixel size smaller than 50 μm and mass accuracy better than 5 ppm has not been achieved yet.

Here, we present developments towards the imaging and identification of proteins at or close to cellular resolution. Data processing remains one of the main challenges for MS imaging in general and for the investigation of tryptic peptides in particular. There are no commonly agreed criteria for the identification of peptides/proteins in MS imaging. The data of our study can be downloaded in the common data format imzML [45] and viewed in open-source software, as described recently [46], in order to be evaluated and reprocessed by alternative approaches. With this study and the publication of the corresponding mass spectral data set as “open data,” we would like to contribute to a more effective discussion and the development of new approaches for tryptic peptide identification in MALDI imaging.

## Material and methods

### Chemicals

Water (HPLC grade), trifluoroacetic acid (TFA), 2,5-dihydroxybenzoic acid (DHB), formic acid (FA), and Eukit were purchased from Fluka (Neu Ulm, Germany). Carboxymethylcellulose (CMC) sodium salt was obtained from Sigma Life Science (MO, USA). Acetic acid (Suprapur), acetonitrile, xylene, and ethanol (Uvasol) were purchased from Merck KGaA (Darmstadt, Germany). Ammoniumbicarbonate (ReagentPlus™), dithiothreitol, iodoacetamide, eosin Y, and hematoxylin were obtained from Sigma-Aldrich Chemie GmbH (Steinheim, Germany). Trypsin was purchased from Promega (Sequencing Grade Modified Trypsin, Madison, WI, USA). All chemicals used in this study were of the highest purity available.

### Tissue samples

Mouse brain tissue and whole body section originating from male C57BL/6 mice were provided by the Institute of Anatomy and Cell Biology, Justus Liebig University, Giessen, Germany. The mouse brain was removed and frozen at − 80 °C after the mouse had been killed.

A microcryotome (HM 525 cryostat, Thermo Scientific, Dreieich, Germany) at − 23 °C was used to obtain sections of 20 μm thickness for mouse brain and 30 μm for the whole mouse body. Only the whole mouse body sample was embedded in CMC (4%) to achieve high-quality sections without distortion of the histological structures. All samples were stored directly at − 80 °C before use. Prior to further sample preparation, the sections were kept in a desiccator to prevent condensation on the surface of the tissue, and optical images of the tissue sections were taken using an Olympus BX-40 microscope (Olympus Europa GmbH, Hamburg, Germany).

### On-tissue trypsin digestion

A series of washing steps were applied to tissue sections for fixation and to remove salts and lipids: after being submersed twice in 70% ethanol for 30 s and 100% ethanol for 15 s, the sample was washed in a solution of 90% ethanol, 9% glacial acid, and 1% water. The glass slide was dipped in water for 2 s to remove the acid in order to obtain optimal conditions for the trypsin application [47]. Trypsin (0.05 μg/μL in 10 mM NH_4_HCO_3_) was deposited with a homebuilt spraying device. Fifteen cycles of 2 μL (flow rate 12 μL/min) for mouse brain and 15 cycles of 15 μL (flow rate 30 μL/min) for the whole mouse body were sprayed. The tissue section was placed for 10 min in a cell culture incubator (37 °C, 100% relative humidity) between spraying cycles and was incubated overnight after the last spraying step.

### MALDI MS imaging

MALDI matrix (30 mg/mL 2,5-DHB in acetone:water 1:1 + 0.1% TFA) was then applied with a homebuilt pneumatic spraying device. The homogeneity and crystal size (5–15 μm) of the matrix were checked with a microscope. In the case of the whole body mouse section, a SunCollect (SunChrome, Friedrichsdorf, Germany) sprayer was used with 10 μL/min and 15 cycles. The MALDI MS imaging experiments were performed with 25 or 50 μm pixel size using an atmospheric pressure imaging ion source (AP-SMALDI10, TransMIT GmbH, Giessen, Germany) attached to an orbital trapping mass spectrometer (Q Exactive, Thermo Fisher Scientific GmbH, Bremen, Germany) [48, 49]. The ion source was equipped with a nitrogen laser with a wavelength of *λ* = 337 nm. The mass spectrometer was operated in positive ion mode at a mass resolution of 35,000–140,000 at *m/z* = 200 over a mass range of *m/z* = 600–1200 or 400–1600. Internal calibration was performed using *m/z* = 993.11210 and a matrix cluster signal as a lock mass, resulting in mass accuracy of better than 3 ppm.

### MS image generation

Selected ion images of known peptide masses were generated with a bin width of Δ*m/z* = ±0.005 using the software package MIRION [50] or MSI Reader [51] after conversion to the common data format for MS imaging: imzML [45]. Intensity values in the ion images were normalized to the highest intensity measured for each ion species separately. Apart from that, images represent raw data without pixel-wise normalization, spatial interpolation, or smoothing.

### Histological staining

The section measured was stained after measurement for a comparison with histological features. An adjacent section was used for staining in some cases. Hematoxylin and eosin (H&E) staining was performed after removing the matrix with 100% ethanol. Tissue sections were stained with Luxol Fast Blue to indicate the distribution of myelin.

### LC-ESI-MS/MS and database search

The workflow for in-solution digestion, LC-ESI-MS/MS detection and database search, which is briefly described in the following, is based on protocols that we have published previously [33, 52]. After homogenization of samples, 5 mM dithiothreitol (57 °C for 45 min) and 15 mM iodoacetamide were added. Trypsin was applied in a 1:30 enzyme/protein (*w*/*w*) ratio, and the sample was incubated for 15 h at 37 °C. An additional purification step using C18 Zip Tips (Varian, Lake Forest, California, USA) was applied,

A nano-LC system (LCPackings/ Dionex, Idstein, Germany) was used for separation of tryptic peptides. Following prefocusing on a trap column, peptides were separated on a C18 capillary column (3 μm particle size, 75 μm inner diameter, 15 cm length) at a flow rate of 300 nL/min. The solvent gradient of water with 2% acetonitrile/0.1% formic acid (*v*/*v*) and acetonitrile with 20% water/0.1% formic acid (*v*/*v*) is described in [52].

The nano-LC was attached to a nanoelectrospray interface of a Q Exactive mass spectrometer (Thermo Fisher Scientific GmbH, Bremen, Germany) operated in positive mode (mass resolution *R* = 70,000 at *m/z* = 200, lock mass: polysiloxane *m/z* = 445.12003). The ten most intense peaks from full scan with a mass resolution of *R* = 70,000 (at *m/z* = 200) were used for fragmentation. Higher-energy collision dissociation (HCD) was used with a normalized collision energy of 28%, isolation window for the precursor: *m*/*z* ± 2.0, mass resolution *R* = 17,500 (at *m/z* = 200). Each of the samples was measured three times and the data were combined for data analysis.

LC-ESI-MS/MS data were processed with Proteome Discoverer software version 1.4 (Thermo Fisher Scientific GmbH, Bremen, Germany) and searched against the UniProtKB database filtered with *Mus musculus* (accessed on 1 July 2013) with the following parameters: mass tolerance for precursor ions, 4 ppm; fragment mass tolerance, 0.02 Da, up to two missed cleavages. Final filtering of results was performed with a maximum peptide mass deviation of 3 ppm and with “high confidence,” (corresponding to a *q* value < 0.01 and a false discovery rate (FDR) of 1%). Two distinct peptides were required for identification of a protein in these LC-MS/MS experiments.

## Results and discussion

### MALDI MS imaging of a coronal mouse brain section at 50 μm pixel size

**Fig. 1:**
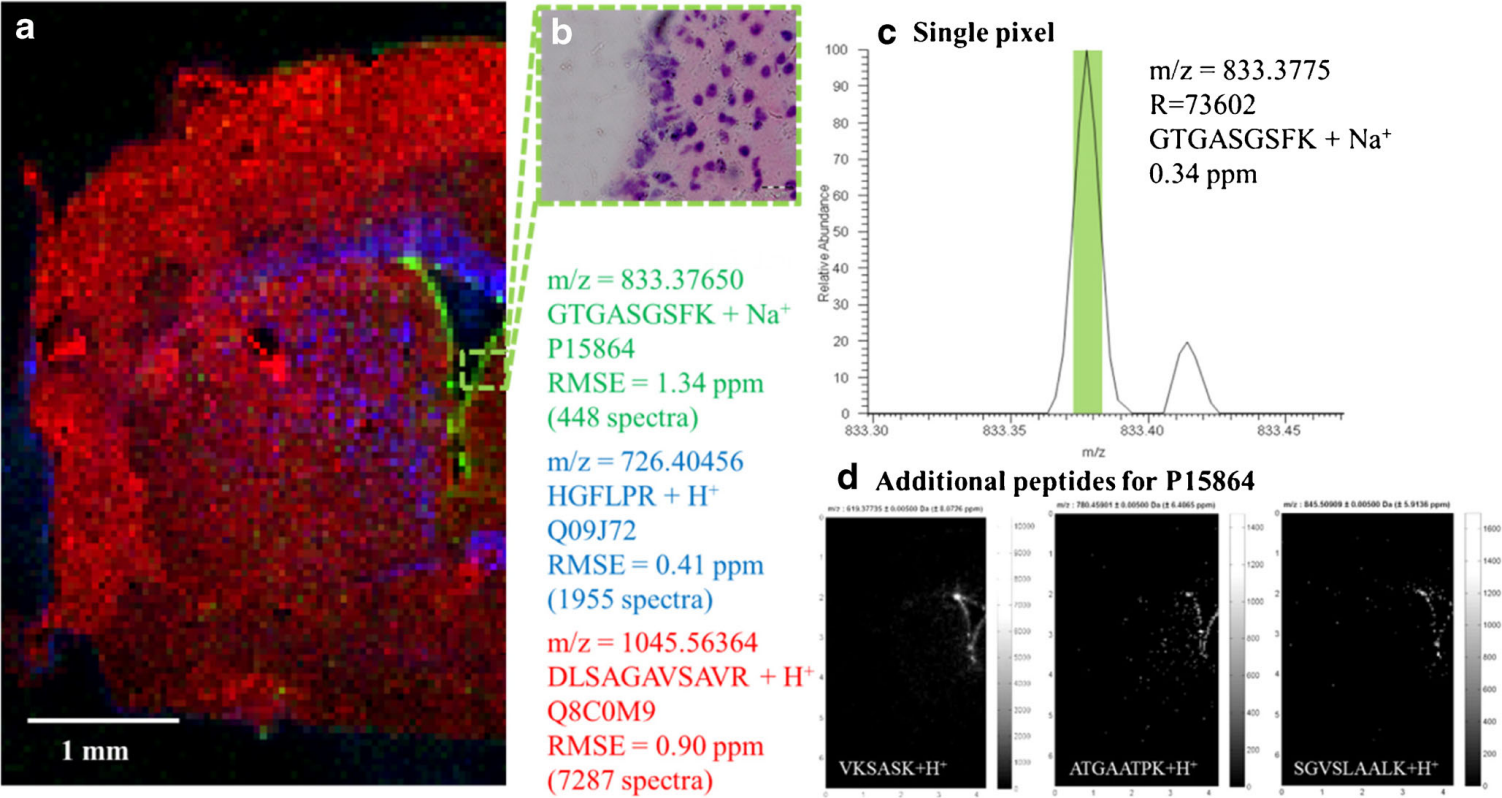
MSI of tryptic peptides in a coronal mouse brain section, imaged with 50 μm pixel size (85 × 135 pixels) and a mass resolution of 140,000 (at *m*/*z* = 200). **a** Overlay of selected ion images of tryptic peptide GTGASGSFK + Na^+^ (green) corresponding to histone 1 protein (P15864), tryptic peptide HGFLPR + H^+^ (blue) corresponding to myelin basic protein (Q09J72), and tryptic peptide DLSAGAVSAVR + H^+^ (red) corresponding to the protein isoaspartyl peptidase/l-asparaginase (Q8C0M9). **b** Magnification of the ependyma area, H&E stained. **c** Mass spectrum of a single pixel (50 μm pixel size) of tryptic peptide GTGASGSFK + Na^+^ corresponding to histone 1 protein (P15864) which is located in ependyma, consisting of one to two cell layers. **d** Additional tryptic peptides for P15864

A coronal mouse brain section (Fig. 1) was imaged with a pixel size of 50 μm (85 × 135 pixels corresponding to 4250 × 6750 μm^2^). A mass resolution of *R* = 140,000 at *m/z* = 200 was used. The tryptic peptide HGFLPR + H^+^ (*m/z* = 726.40456 ± 0.005) corresponding to myelin basic protein is shown in blue in Fig. 1a. It shows high intensities in the *corpus callosum* and *caudoputamen*, which corresponds well to the fact that myelin basic protein is mainly located in the white matter of the brain as part of the myelin sheath. All images in this study were generated with a bin size of ∆*m/z* = ± 0.005 and without interpolation or normalization. Tryptic peptide DLSAGAVSAVR + H^+^ (*m/z* = 1045.56364 ± 0.005) corresponding to the protein isoaspartyl peptidase/l-asparaginase showed a complementary distribution and was primarily detected in the cortex area (Fig. 1a, red).

This measurement also revealed more detailed histological structures. This includes the epithelial lining (*ependyma*) of the lateral ventricle, which consists of only one or two cell layers, as shown in Fig. 1b (H&E-stained tissue section after measurement). The *ependyma* is clearly defined in the MS image by the tryptic peptide GTGASGSFK + Na^+^ (*m/z* = 833.37650 ± 0.005) corresponding to a histone 1 protein (Fig. 1a, green). The mass peak of this peptide obtained from a single 50 μm pixel is shown in Fig. 1c (mass resolution *R* = 73,602 and mass deviation 0.34 ppm). The overall mass accuracy for this peptide, as calculated from 448 individual spectra in this imaging measurement, was 1.34 ppm (root mean square error, RSME). This demonstrates the high mass accuracy over the whole measurement, which was also confirmed for the other peptides shown in Fig. 1a (RMSE values of 0.41 ppm and 0.90 ppm, respectively). The spatial distribution of histone 1 was confirmed by MS images of 14 additional tryptic peptides that correspond to this protein (see Electronic supplementary material (ESM) Fig. S1), of which three examples are shown in Fig. 1d. Additional MS images of peptides for the other proteins shown in Fig. 1a are displayed in the ESM Figs. S2 and S3. Details for identification of proteins are discussed in the next section. These are the first results for MS imaging of tryptic peptides that show a single (or double) cell layer.

An important aspect of MS imaging experiments, especially if on-tissue chemistry is onvolved, is reproducibility. We imaged three neighboring mouse brain sections at 50 μm pixel size. Examples of tryptic peptides of myelin basic protein are shown in ESM Fig. S4. These experiments resulted in comparable MS images and, thus, confirmed the reproducibility of our protocol.

### Identification workflow

**Fig. 2:**
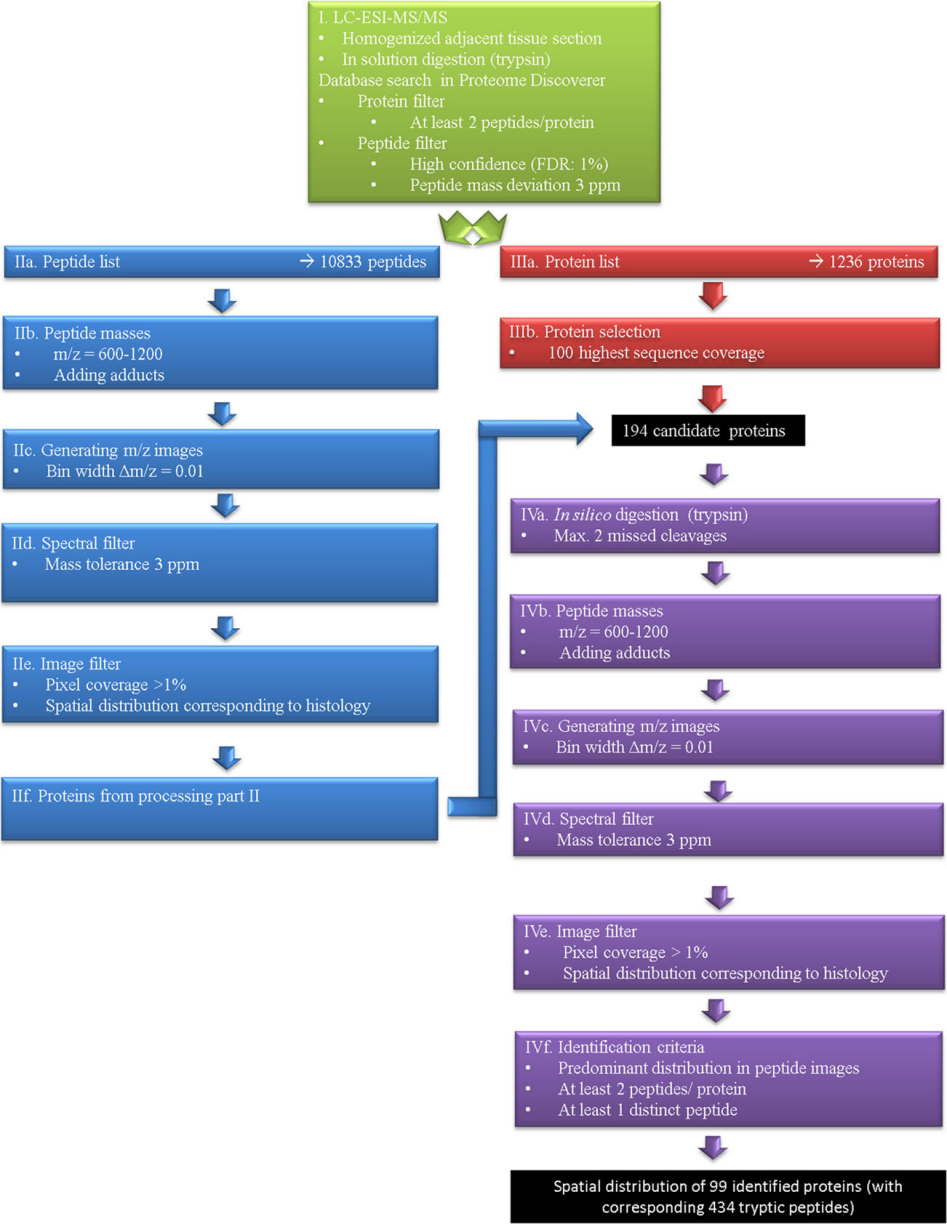
Workflow for tryptic peptide identification in MALDI MS imaging experiments with example numbers of the measurement shown in Fig. 1 (coronal mouse brain section with 50 μm pixel size). Part I consists of LC-MS/MS measurements to identify proteins that are present in the analyzed tissue. Parts II and III have the purpose to select suitable candidate proteins for in silico digestion. The identification of proteins in part IV is independent and can also be performed with alternative protein candidates. In this example, about 50% of proteins were identified with at least four corresponding peptides

Peptides shown in Fig. 1 are only a small part of the signals detected in the mouse brain tissue section. Identification of proteins after on-tissue digestion in MS imaging experiments is a complex process and there is no generally agreed procedure yet. It is not sufficient to only provide a list of supposedly identified proteins. Instead, the approach of identification and selection criteria should be reported as well. We have considered different methods (including direct comparison and in silico digestion) and finally decided on a combination of approaches, as discussed in the following. Our identification workflow for MALDI MS imaging of tryptic peptides is based on a comparison with the LC-MS/MS measurements of a homogenized adjacent section, as in our previous work [33]. We augmented this approach in the current study with in silico digestion of proteins and the inclusion of adduct ions. A scheme of our workflow is shown in Fig. 2. After a database search of LC measurements (part I), two different processing pathways were used to select proteins for in silico digestion: one based on *peptides* which were detected in the LC-MS/MS measurement (part II), and one based on *proteins* identified by a database search of LC-MS/MS measurements (part III). Images of the resulting tryptic peptides were filtered by different parameters and assigned to corresponding proteins (part IV). The details of this procedure are discussed in the following.

Part I of the data processing was the database search of LC-MS/MS data with strict parameters (3 ppm mass deviation, 1% FDR and at least two peptides per protein) resulting in the identification of 10,833 peptides corresponding to 1236 proteins. It is not practical to use 1000+ proteins for in silico digestion and subsequent comparison with MS imaging data, therefore, we applied two methods to select candidate proteins (parts II and III of our workflow).

In processing part II, the peptide list (10,833 peptides) was compared directly with MS imaging experiment data in analogy to our previous approach [33]. In addition to protonated peptides, sodium, potassium, and ammonium adducts were included in the data analysis as they were observed in significant numbers in previous experiments using our method. Inclusion of these adducts resulted in a total of 6822 peptide masses in the mass range *m/z* 600–1200 (IIb). In the next step (IIc), images for all of these peptide masses were generated with a bin width of Δ *m/z* = ± 0.005. Centroided masses for the entire image of each peptide mass were calculated and a filter of a maximum mass deviation of 3 ppm (IId) was applied in order to avoid false positive assignments. A minimum pixel coverage of 1% was applied to the remaining 5942 images in order to exclude MS images with no or very limited spatial information (IIe). While these steps eliminated possible false-positive assignments and also MS images with a very low number of pixels, still a large number of images with no discernable structure (noise) or a distribution that corresponds to the background (glass slide) remained. Manual inspection revealed that 134 peptide masses showed a spatial distribution that was correlated with the histological structure of the tissue section measured. These peptides were assigned to proteins based on the result of the database search (part I). This resulted in MS images of peptides that correspond to 118 proteins (IIf).

In addition to the proteins selected in part II, we also included 100 proteins which showed the highest sequence coverage in the database search of the LC-MS/MS measurement (as a rough indication of high abundance in the analyzed mouse brain tissue) (part III). These abundant proteins were chosen as candidates because they are also more likely to be detected by MS imaging. Combining part II (118 proteins) and part III (100 proteins) resulted in 194 different candidate proteins (22 proteins were present in parts II and III, and 2 proteins were excluded because they turned out to be of human origin on a closer inspection of the database search results).

Processing part IV: these 194 proteins (a complete list can be found in ESM Table S4) were used as candidate proteins and were subjected to in silico digestion (IVa). This procedure resulted in 26,125 theoretical peptide masses in the mass range *m/z* 600–1200 (IVb), including the hydrogen, sodium, potassium, and ammonium species. These peptide masses were treated in analogy to the peptide list in part II.

Images were generated with a bin width of Δ *m/z* = ± 0.005 U (IVc) and peptide masses were filtered by mass accuracy (RSME < 3 ppm) in step IVd (20,006 images). A total of 1240 of these peptide images showed a pixel coverage > 1% and a spatial distribution corresponding to the histology of the tissue section measured (IVe). We compared MS images of peptides that correspond to the same protein and considered a protein identified when more than 50% of the images showed matching distributions (“predominant distribution”). Additional requirements for identification (IVf) included the presence of at least two peptides per protein as well as the identification of at least one distinct peptide (only assigned to one protein of our data set). After filtering the data with these criteria, we obtained the spatial distribution of 99 proteins (with 434 corresponding peptides).

Sixty-nine of the resulting proteins originated from part II (peptide list), twenty-one from part III (protein list), and nine were included in both pathways. Thus, the more laborious approach using the peptide list (part II) yielded a higher number of proteins identified. It should be noted that parts II and III have the purpose to select suitable candidate proteins for in silico digestion. The identification of proteins in part IV is independent and can also be performed with alternative protein candidates.

In our case, almost 50% of proteins were identified with four or more corresponding peptides and 30% of proteins were identified with three peptides. Most peptides were detected as protonated ions; however, a significant number of sodium adducts and potassium and ammonium adducts were also detected. This is in contrast with most studies published about on-tissue digestion MS imaging and is most likely due to differing conditions in our experiment (e.g., DHB as matrix, atmospheric pressure ionization). More than 70% of all proteins identified exceeded 25 kDa (see ESM Table S5) and thus, would not be detected in regular intact protein imaging experiments. In addition to myelin, 30 proteins were found which are located in the *corpus callosum* and *caudoputamen*, 14 proteins showed the highest intensities in the *ependyma* and 54 proteins were distributed predominantly in the *cortex* of the mouse brain (see ESM Table S5). It should be noted again that we applied very strict criteria in selecting these proteins. More relaxed filters (e.g., only one peptide per protein as applied in some previous studies) would result in a significantly higher number of proteins identified (also see the discussion in our previous study on the identification of peptides after on-tissue digestion [42]).

It should be noted that on-tissue digestion also results in some limitation. For example, it does not always allow for detection of post-translational modifications or the distinction of isoforms. One example is peptide *GTGASGSFK* in Fig. 1 (green) which is assigned to “histone 1.2” in our workflow (based on the LC-MS database search). As a differentiation of different histone 1 isoforms is not possible, we report this protein as “histone 1” (see also ESM Fig. S1). Irrespective of these limitations, on-tissue digestion can greatly expand the capabilities of MS imaging for protein identification.

Reporting guidelines for MS imaging in general have been proposed recently [53], but there is no generally agreed approach for the identification of proteins after on—tissue digestion yet. Results for proteins are often reported with only one peptide per protein and very limited information on the identification process at all. We do not propose our approach as a final result, but rather see it as an intermediate step towards a generally accepted method for the identification of proteins in on-tissue digestion experiments. In order to facilitate this process, we have made our data set available in the public repository PRIDE so that other groups can use it as a test case for their own (software-based) approaches and compare it with our results. The data set is available via ProteomeXchange (http://www.proteomexchange.org/) with identifier PXD003172. The data can be downloaded in the common data format imzML [45] and viewed in open-source software, as described recently [54]. If this data set is used, it should be taken into account (a) that peptides are often detected as sodium/potassium adducts and (b) that the spectra include a number of high intensity matrix peaks (which can be excluded from data analysis based on accurate mass analysis).

### MSI of mouse brain with a pixel size of 25 μm

**Fig. 3:**
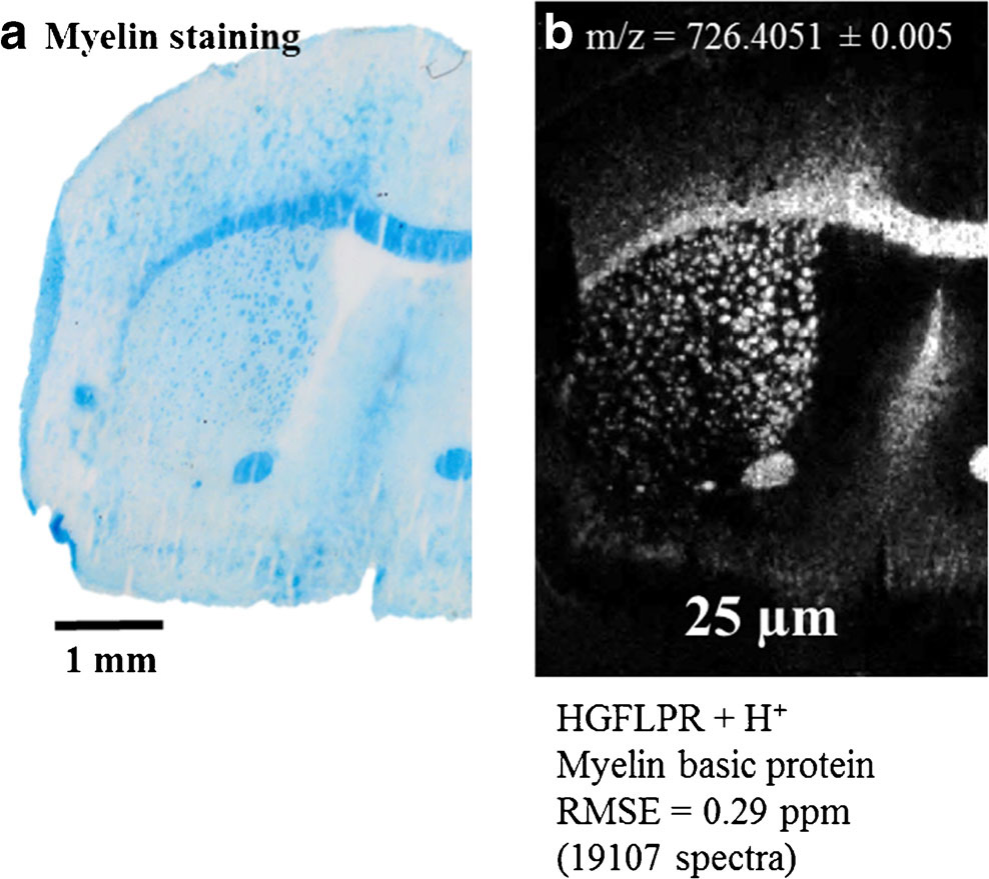
MS imaging of coronal mouse brain section. **a** Myelin staining of adjacent tissue section. **b** Selected ion image with a pixel size of 25 μm of tryptic peptide HGFLPR + H^+^ (*m*/*z* = 726.40456 ± 0.005) corresponding to myelin basic protein

Our sample preparation protocol can also be used for a higher lateral resolution. The effect of the improved spatial resolution using our method is displayed in Fig. 3, which shows a measurement of a coronal mouse brain section with 25 μm pixel size (180 × 250 pixels). It was measured with a mass resolution setting of *R* = 35,000 (at *m/z* = 200).

The ion image selected in Fig. 3b shows the myelin peptide HGFLPR + H^+^ (*m/z* = 726.40456 ± 0.005) with a clear spatial distribution and a good correlation to the myelin-stained adjacent section (Fig. 3a). The *corpus callosum* is well defined and smaller structures in the *caudoputamen* area are clearly visible. Despite the smaller pixel size, the mass accuracy is still very high over the whole measurement (RMSE = 0.29 ppm, 19,107 spectra of the peptide shown in Fig. 3b). In comparison with the measurement of a comparable tissue section with 50 μm pixel size (Fig. 1), more than 90% of the 434 peptides assigned were also found in the 25 μm measurement (and show the same spatial distribution). Examples are shown in the ESM (Figs. S5 and S6).

**Fig. 4:**
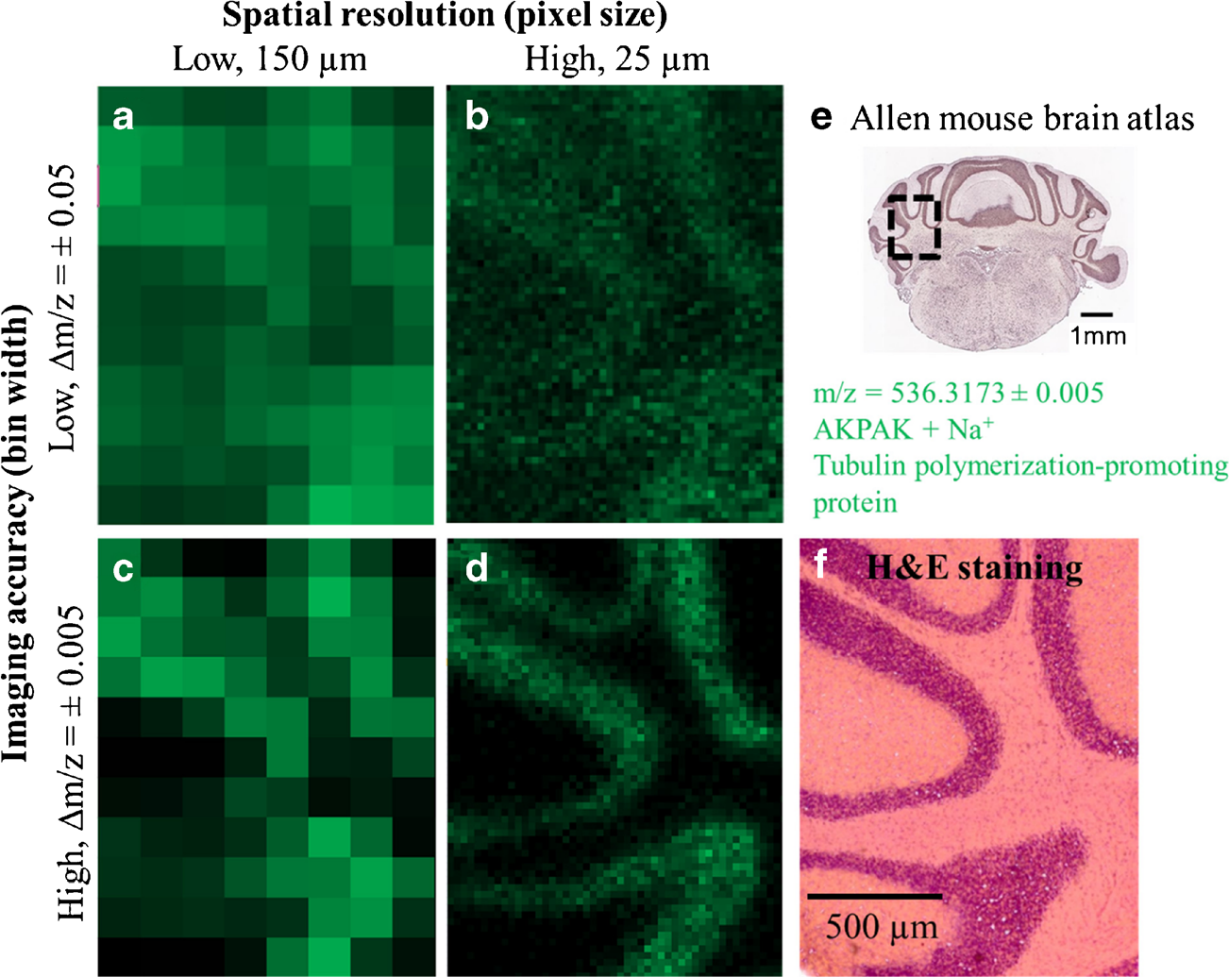
Influence of spatial resolution and imaging accuracy in a measurement of mouse brain cerebellum with 25 μm pixel size shown on *m*/*z* = 536.316703 (tryptic peptide of tubulin polymerization-promoting protein). **a**–**c** selected ion images recalculated for different acquisition parameters. **d** Selected ion image measured with 25 μm pixel size and generated with a small bin width of Δ*m*/*z* = ± 0.005 U. **e** ALLEN mouse brain atlas: in situ hybridization of tubulin polymerization-promoting protein. **f** H&E staining of an adjacent section

Another measurement at 25 μm pixel size of a different area of the mouse brain is shown in Fig. 4. The cerebellum region was measured with 50 × 65 pixels and a mass resolution of *R* **=** 70,000 (at *m/z* = 200). The ion image selected (Fig. 4d) of the tryptic peptide AKPAK + Na^+^ corresponding to a tubulin polymerization-promoting protein with a bin width of Δ*m/z* = ± 0.005 (*m/z* = 536.316703 ± 0.005) correlates to the structure of the granular layer, which is shown in the H&E-stained image in Fig. 4f. Figure 4a–d illustrates the main advantage of our approach, i.e., the combination of high mass resolution and high spatial resolution in a single experiment. Figure 4c shows a recalculated image at a pixel size of 150 μm, which corresponds to the typical spatial resolution for MALDI MS imaging experiments of tryptic peptides. As expected, the detailed structure of the granule cell layer (*stratum granulosum*) is no longer visible. However, high spatial resolution alone is not sufficient to resolve these histological features. Figure 4b shows an image with 25 μm pixel size but recalculated with a bin width of Δ*m/z* = 0.1, resembling the imaging accuracy of well calibrated MALDI-TOF measurements. This lower imaging accuracy leads to an overlap of neighboring mass peaks (see ESM Fig. S7) and, thus, results in a loss of spatial information about the peptide. Predictably, the result contains even less information in experiments with low spatial resolution and low imaging accuracy (Fig. 4a). Similar effects were observed for a large portion of peptides identified in our experiments. It can be derived from our results that highly specific and detailed images of tryptic peptides are only possible if spatial resolution of several tenths of a micrometer and mass accuracy in the low ppm range are combined within one experiment.

The optical image (measured section), myelin staining (adjacent section) and an overlay of three different tryptic peptides, including peptides corresponding to myelin basic protein, are shown in ESM Fig. S8, along with an H&E-stained section (adjacent section). This figure corresponds to the “Online abstract figure.”

Figure 4e shows in situ hybridization data representing mRNA levels for a tubulin polymerization-promoting protein in a coronal mouse brain section obtained from the *Allen mouse brain Atlas* [55]. The localization of this protein in the granular layer of the cerebellum correlates very well with the MS imaging results of the corresponding peptide (Fig. 4d). This data was obtained by an independent method and, thus, offers a valuable possibility to confirm the spatial distribution data obtained by MS imaging in mouse model tissue. However, it should be noted that there might be a discrepancy between the mRNA level and protein expression level [56]. Moreover, not all proteins are present in that database and also not every orientation, meaning that not every anatomical plane is available.

### MSI of tryptic peptides in mouse whole body section

**Fig. 5:**
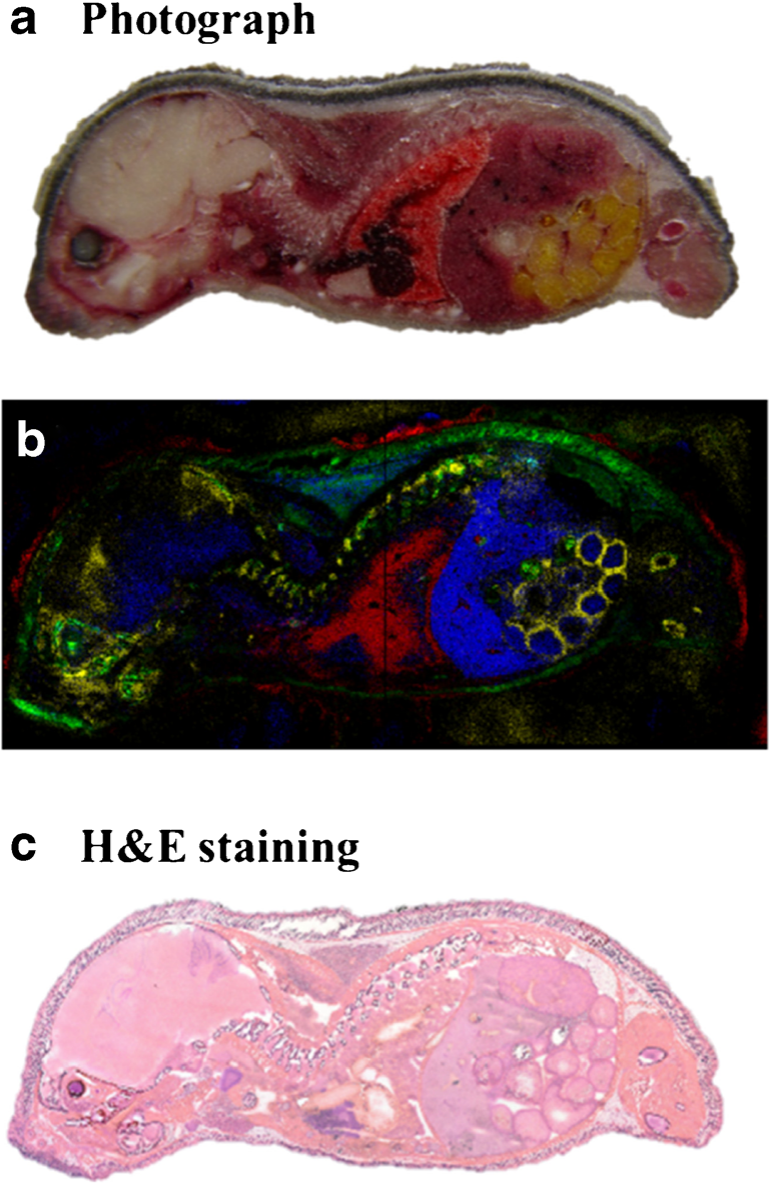
Whole mouse body section. **a** Photograph of block-face embedded in CMC during cutting process. **b** MALDI MS image: overlay of selected ion images of *m*/*z* = 1045.56365 ± 0.005 (blue), *m*/*z* = 519.32492 ± 0.005 (green), *m*/*z* = 530.25862 ± 0.005 (yellow), and *m*/*z* = 612.28662 ± 0.005 (yellow). **c** H&E-stained section after measurement

Our initial work has been focused on the analysis of mouse brain tissue as a model system. However, optimum conditions for enzymatic digestion and data acquisition depend strongly on the tissue properties (e.g., lipid content). Therefore, our sample preparation protocol was evaluated for different sample types. A very efficient pathway to evaluate a protocol for a variety of tissue types in one experiment are whole body sections of animal models, as shown in Fig. 5. A whole body section of a 6-day-old mouse was measured with a pixel size of 50 μm (720 × 330 pixels) and a mass resolution of *R* = 70,000 at *m/z* = 200 (Fig. 5b). Different potential tryptic peptide masses were detected which were located specifically in individual organs and showed good correlation to the block-face image during sectioning (Fig. 5a) and the H&E-stained section (Fig. 5c). The H&E staining was carried out on the same section after the MS measurement. Ten example proteins, obtained from part II of our identification workflow (see Fig. 2), were digested in silico for the whole mouse body*.* It was not possible to identify proteins reliably with our identification protocol. In contrast to the mouse brain sections discussed above, tryptic peptides of all proteins in the whole body section show four to five different distributions. This is due to the much higher complexity (higher number of different proteins) of the tissue. Reliable identification of these proteins would require more detailed LC measurements for different organs of the mouse. Another possibility is to use LESA for more detailed LC measurements [43] or smaller sections for in-solution digest [57]. Nevertheless, this dataset includes important information and we choose to show examples of tryptic peptides and indicate possible corresponding proteins. An overlay of four tryptic peptides of four different proteins is shown in Fig. 5b. The ion image selected of *m/z* = 519.32492 ± 0.005 (Fig. 5b, green) could be related to the tryptic peptide NIGAK/NQLK + NH_4_^+^ corresponding to a 60-kDa heat shock protein (P63038, 60.9 kDa), which is mainly located in the spine and the skin. For the selected ion image of *m/z* = 530.25862, a possible tryptic peptide VFDK + Na^+^ of myosin light chain 1/3, skeletal muscle isoform (P05977, 20.5 kDa) was found in the spine and brain (especially *hippocampus* and *cerebellum* region: yellow). It also shows a highly structured distribution in the intestines. The tryptic peptide GRGITGIEDK + H^+^ (*m/z* = 1045.56365 ± 0.005), shown in blue, tentatively assigned to transketolase (P40142, 67.6 kDa), is mainly distributed in the midriff and liver with weak signals in the lung, thymus and brain. The red color displays the *m/z* = 612.28662 ± 0.005 of the possible tryptic peptide GKQGGK + K^+^ of histone H2A (A0AUV1,13.8 kDa) which can be found in the lung region and the outer part of the skin.

The data for this 90-h experiment were acquired in four separate files of about 10 GB each. A total of 230,000 spectra were acquired. Mass spectral quality (resolution and accuracy) was comparable with the mouse brain measurements discussed above. It is remarkable that, despite the long acquisition time, the signal intensity was stable over the whole area without image normalization. Thus, we achieved stable conditions for digestion and measurement at 50 μm pixel size over an area of 36 × 16.5 mm. This measurement demonstrates that our current protocol is suitable for larger areas (several centimeters) and different tissue types.

### Data processing for large files: imzML

**Fig. 6:**
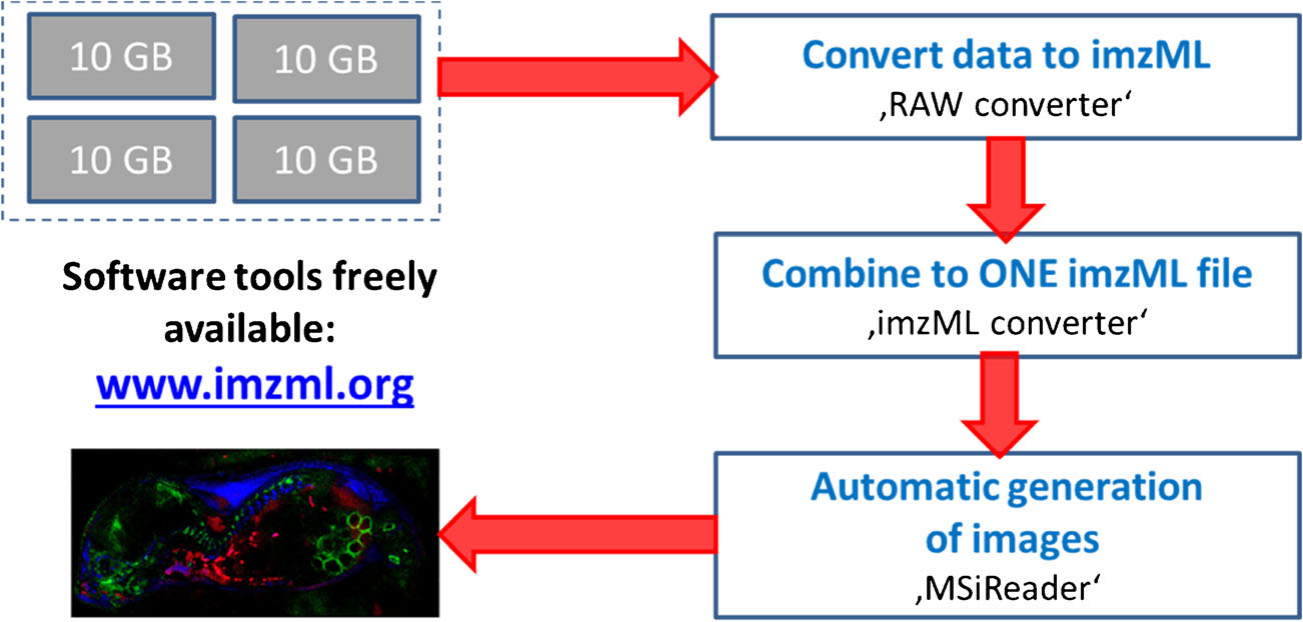
Workflow of processing MS imaging data using imzML as an exchange data format. Different software tools can be applied sequentially for more flexible data handling. See text for more details on the software tools applied

The large amount of data generated in the whole body mouse experiment (ca. 40 GB) required a dedicated workflow for efficient data analysis and MS image generation. Therefore, the original data files were processed using the common data format for MS imaging imzML [45]. This data format provides the possibility of choosing from a pool of different (complementary) software tools. This greatly enhances the flexibility for data processing and often speeds up data analysis significantly. In this example, a sequential workflow of different software tools was used to generate the MS image shown in Fig. 5. The workflow of stitching and processing these large files is described in Fig. 6. In a first step, the individual Thermo RAW data files (about 10 GB each) were converted to imzML using a “RAW converter” developed in our group [45]. Subsequently, these files were combined into one imzML using the “imzML converter,” developed at the University of Birmingham (UK) [58]. The MS images were generated automatically based on a list of potential tryptic peptides with the open-source software “MSiReader,” developed at North Carolina State University (Raleigh, NC, USA) [51].

This procedure was necessary in order to process and analyze the large data set (> 40 GB) which was originally acquired in multiple files. Using this workflow, several hundred MS images can be automatically generated in 1 h, whereas the previous approach required about 20 min for a single image. This workflow is based on tools which are freely available through the website www.imzml.org and can, thus, be applied by other groups as well.

## Conclusions

This study reports on a number of improvements for the analysis of proteins after on-tissue digestion. Histological features that consist of one to two cell layers could be imaged and showed excellent correlation with the optical image. This kind of detail has not been shown for MS imaging of tryptic peptides before. We also demonstrate a spatial resolution of 25 μm pixel size for tryptic peptides. The number of peptides detected was comparable with the 50 μm pixel measurement, indicating that detection sensitivity is not the main limiting factor in our current method, i.e., the reduction in tissue material that is desorbed/ionized (pixel area is four times smaller) does not significantly reduced spectral quality. Consequently, pixel sizes below 25 μm are possible from the point of the ionization process. Instead, spatial resolution is currently limited by analyte delocalization caused by the trypsin application procedure, which has to be further optimized in future studies.

Identification remains a major challenge in MS imaging of tryptic peptides/on-tissue digestion. The results provided for these experiments should include more than a list of identified proteins. We developed a new identification workflow that includes multiple steps for reliable identification. We updated our previous approach by now including an in silico digest of candidate proteins which are obtained by complementary LC-MS/MS measurements. This increased the number of proteins identified. In the 50-μm measurement, we identified 99 proteins (434 corresponding tryptic peptides) in mouse brain with strict selection criteria. It should be noted that a more relaxed filtering would result in significantly more proteins. More than 70% of these proteins exceed 25 kDa and would, thus, not have been detected in regular top-down experiments (without on-tissue digest).

In addition to the direct comparison of stained sections, MS images of tryptic peptides can also be compared with data from publicly available resources. We used in situ hybridization data from the Allen mouse brain atlas as a complementary technique to confirm our approach.

The stability and reproducibility of our on-tissue digestion workflow was demonstrated by a triplicate measurement for mouse brain and by a measurement of a whole body mouse section. In the latter case, signal intensities were stable for 90+ h. We described a sequential workflow for handling larger datasets which is based on the conversion to imzML and use of freely available software tools. This dataset shows that the protocol proposed can be applied to a number of different tissue types. With careful optimization of experimental parameters, MS images with a pixel size of 5 to 10 μm and, thus, the detection of single cells seems to be possible in the near future. Further developments in our lab will focus on the automation of the data processing workflow, which we will base on imzML software.

In addition to developments in individual laboratories, activities in the MS imaging community are required to improve MS imaging after on-tissue digestion. The two major challenges remain trypsin (enzyme) application and peptide/protein identification. Multicenter studies are a good way to evaluate and improve MS imaging methods [54]. The second task for the MS imaging community is to define common criteria for the identification of proteins in MS imaging. We see our manuscript as a contribution to this process and have, therefore, made an example data set (50 μm coronal mouse brain section) available as “open data” in PRIDE (imzML data format).

If these combined attempts are successful, MS imaging of proteins after on-tissue digestion can be established as a reliable and routine technique in the coming years.

## Electronic supplementary material

ESM 1 (PDF 3846 kb)
