# Supplementary material for: Approaching cellular resolution and reliable identification in mass spectrometry imaging of tryptic peptides
Source: Anal Bioanal Chem. 2018 Aug 1;410(23):5825–37. doi: 10.1007/s00216-018-1199-z (PMC6096711; doi:10.1007/s00216-018-1199-z)
Supplement: Supplementary file 1 — (PDF 3846 kb) [file 216_2018_1199_MOESM1_ESM.pdf]

Analytical and Bioanalytical Chemistry

Electronic Supplementary Material

Approaching cellular resolution and reliable identification in mass spectrometry imaging of tryptic peptides

Katharina Huber, Pegah Khamehgir-Silz, Thorsten Schramm, Vladimir Gorshkov, Bernhard Spengler, Andreas Römpp

I. MALDI MS imaging of a coronal mouse brain section with 50 µm pixel size

Histone 1 (Histone 1.2, P15864)

As mentioned in the manuscript, isoforms cannot be distinguish after tryptic digestion. The isoform Histone 1.2 is shown here as an example as it was assigned for these peptides in our workflow.

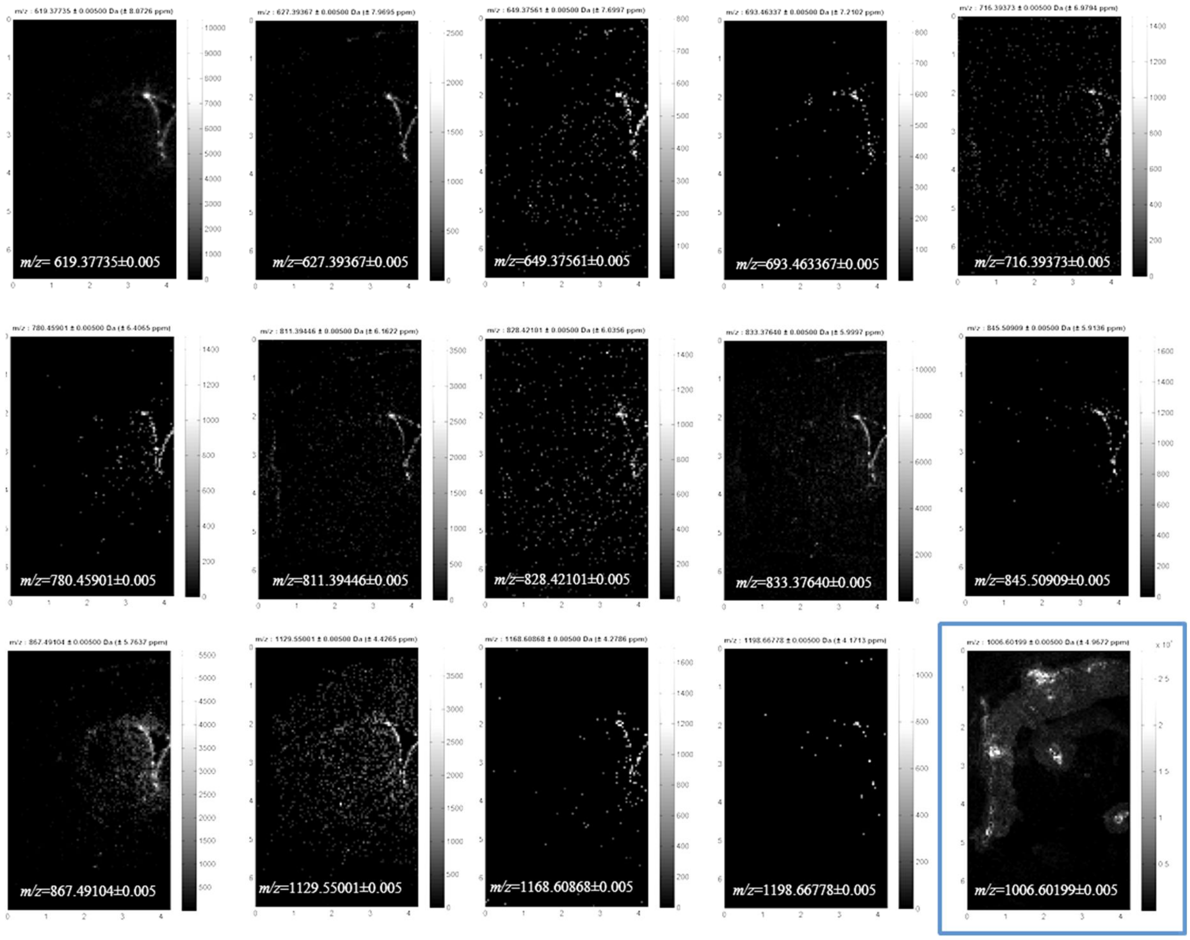

Fig. S1 Additional tryptic peptides corresponding to histone 1.2 and image of peptide mass which doesn't show the *ependyma* structure highlighted in blue

Table S1 Identified tryptic peptides for histone 1.2

m/z_{theo}	adduct	sequence	Δm/z (ppm)
619.37735	H	VKSASK	1.51
627.39367	H	KPAGVR	0.35
649.37561	Na	KPAGVR	0.74
693.46337	Na	IKLGLK	1.75
716.39373	H	ATGAATPK	-0.36
780.45901	Na	GILVQTK	0.91
811.39446	H	GTGASGSFK	0.51
828.42101	NH4	GTGASGSFK	0.75
833.3764	Na	GTGASGSFK	1.00
845.50909	H	SGVSLAALK	1.85
867.49104	Na	SGVSLAALK	-0.25
1129.55001	Na	ALAAAGYDVEK	0.95
1168.60868	K	ERSGVSLAALK	2.07
1198.66778	H	ASGPPVSELITK	0.89

Myelin basic protein (Q09J72)

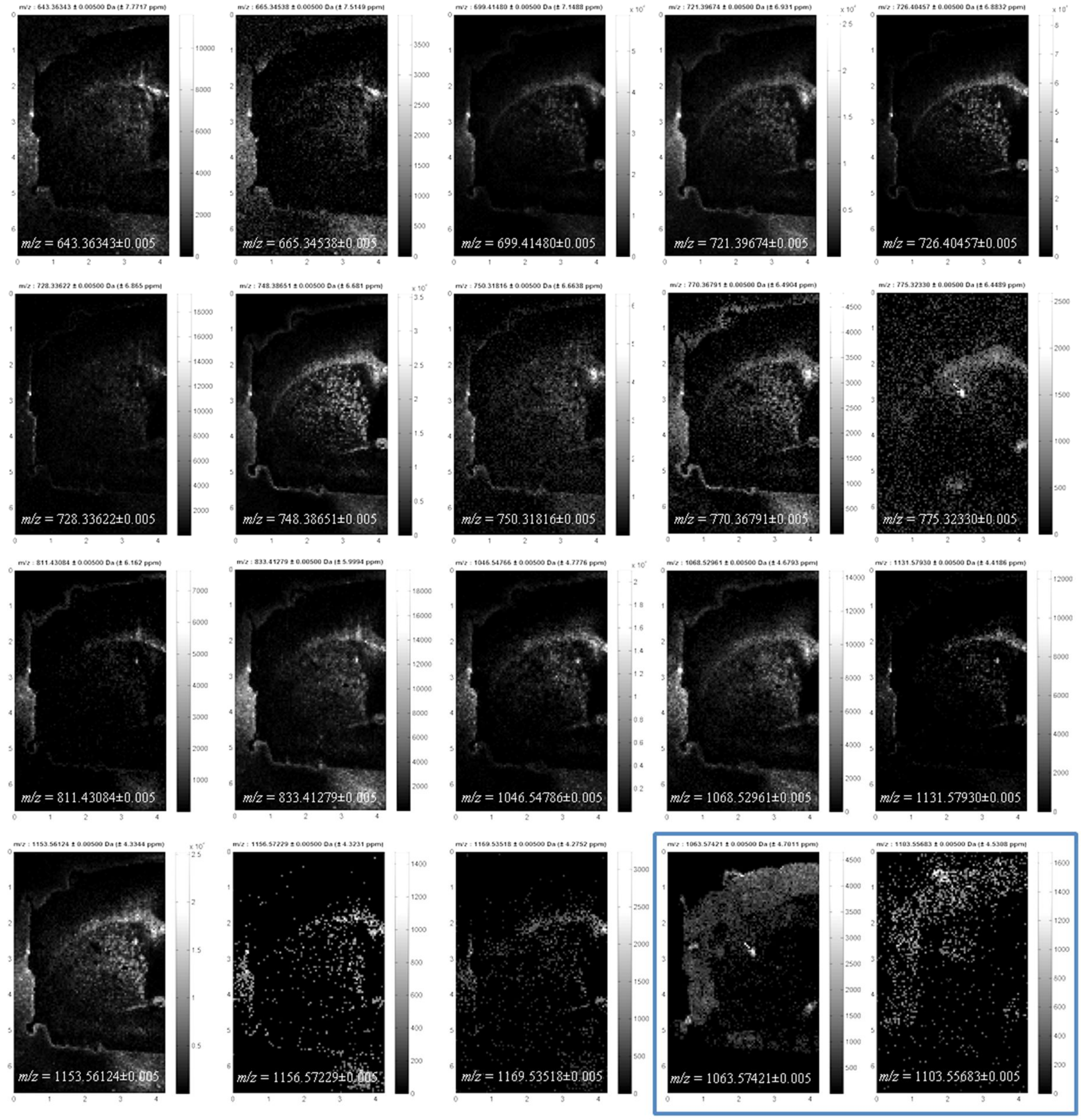

Fig. S2 Additional tryptic peptides corresponding to myelin basic protein and image of peptide mass which doesn't show the *corpus callosum* structure highlighted in blue

Table S2 Identified tryptic peptides for myelin basic protein

m/z_{theo}	adduct	sequence	Δm/z (ppm)
643.36343	H	RPSQR	1.26
665.34538	Na	RPSQR	0.77
699.4148	H	NIVTPR	0.32
721.39674	Na	NIVTPR	0.33
726.40457	H	HGFLPR	-0.38
728.33622	H	FFSGDR	-0.59
748.38651	Na	HGFLPR	0.08
750.31816	Na	FFSGDR	0.32
770.36791	NH4	EDNTFK	1.55

775.3233	Na	EDNTFK	2.52
811.43084	H	TPPPSQGK	1.68
833.41279	Na	TPPPSQGK	0.72
1046.54766	H	DTGILDSIGR	-0.19
1068.52961	Na	DTGILDSIGR	0.69
1131.5793	H	TTHYGSLPQK	1.89
1153.56124	Na	TTHYGSLPQK	1.36
1156.5723	K	RELSAEKASK	-0.84
1169.53518	K	TTHYGSLPQK	-1.49

Isoaspartyl peptidase/L-asparaginase (Q8C0M9)

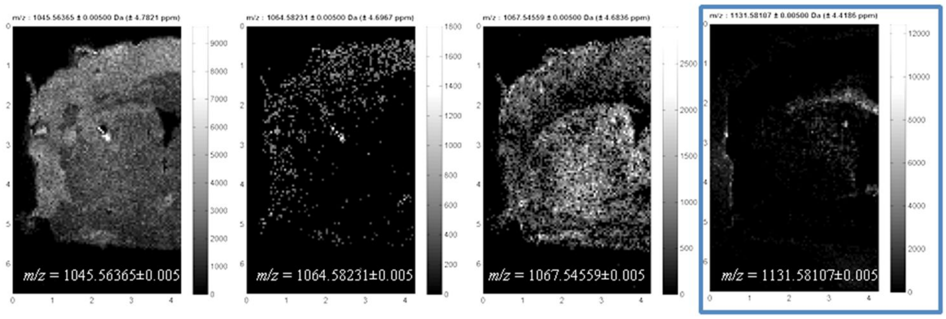

Fig. S3 Additional tryptic peptides corresponding to isoaspartyl peptidase/L-asparaginase and image of peptide mass which doesn't show the *cortex* structure highlighted in blue

Table S3 Identified tryptic peptides for isoaspartyl peptidase/L-asparaginase (Q8C0M9)

m/z_{theo}	**adduct**	**sequence**	**Δm/z (ppm)**
1045.56365	H	DLSAGAVSAVR	-0.30
1067.54559	Na	DLSAGAVSAVR	1.10
1064.58231	Na	ELVREGIAR	-1.44

II. Reproducibility

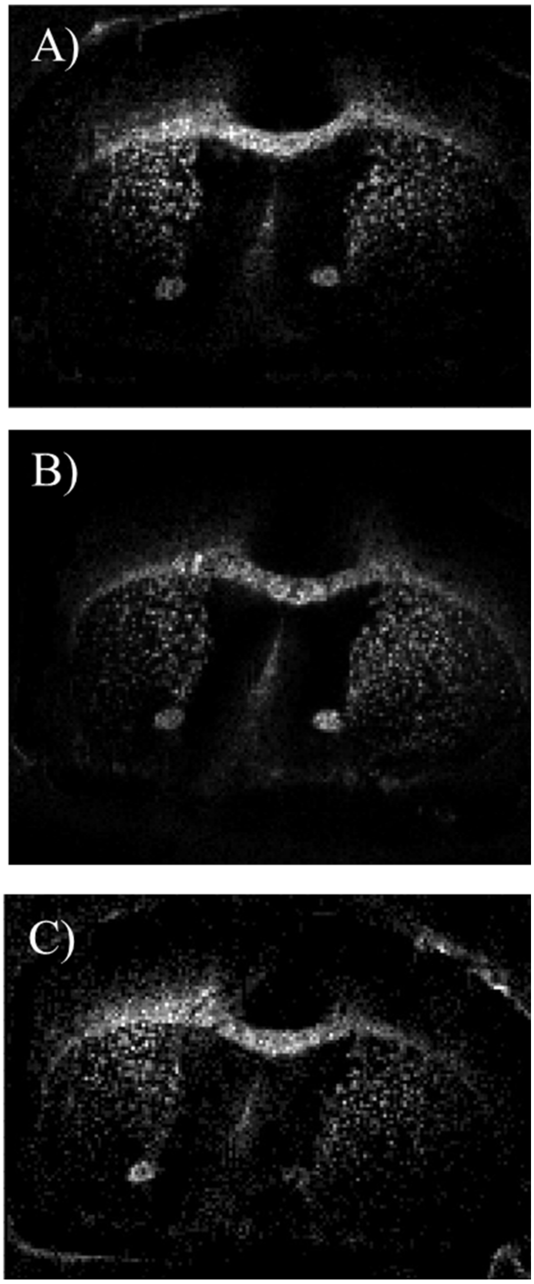

Fig. S4 Reproducibility: Measurements with 50 µm pixel size of adjacent mouse brain sections A) measured with Exactive mass spectrometer B)) measured with Q Exactive mass spectrometer C) measured with Exactive mass spectrometer shown on m/z =726.40456 ± 0.005 of tryptic peptide HGFLPR + H^+ corresponding to myelin basic protein

III. Identification workflow for tryptic peptides in MALDI MS imaging experiments

Table S4 Candidate proteins used for *in silico* digestion

AccNr	Protein names	mass (kDa)	protein origin
A8DUK4	Beta-globin (Protein Hbb-bs) (Protein Hbb-bt)	15.7	Pathway III
B0V2H4	V-type proton ATPase subunit G 2	55.91	Pathway III
G3UWG1	MCG115977 (Uncharacterized protein)	56.19	Pathway III
G3X9L6	MCG55033 (Uncharacterized protein)	77.5	Pathway III
O08553	Dihydropyrimidinase-related protein 2 (DRP-2) (Unc-33-like phosphoprotein 2) (ULIP-2)	62.2	Pathway III
O08599	Syntaxin-binding protein 1 (Protein unc-18 homolog 1) (Unc18-1) (Protein unc-18 homolog A) (Unc-18A)	67.5	Pathway III
O08997	Copper transport protein ATOX1 (Metal transport protein ATX1)	58.82	Pathway III
P00493	Hypoxanthine-guanine phosphoribosyltransferase (HGPRT) (HGPRTase) (EC 2.4.2.8) (HPRT B)	63.3	Pathway III
P00920	Carbonic anhydrase 2 (EC 4.2.1.1) (Carbonate dehydratase II) (Carbonic anhydrase II) (CA-II)	29	Pathway III
P05064	Fructose-bisphosphate aldolase A (EC 4.1.2.13) (Aldolase 1) (Muscle-type aldolase)	39.3	Pathway III
P05201	Aspartate aminotransferase, cytoplasmic (cAspAT) (EC 2.6.1.1) (EC 2.6.1.3) (Cysteine aminotransferase, cytoplasmic) (Cysteine transaminase, cytoplasmic) (cCAT) (Glutamate oxaloacetate transaminase 1) (Transaminase A)	62.71	Pathway III
P05202	Aspartate aminotransferase, mitochondrial (mAspAT) (EC 2.6.1.1) (EC 2.6.1.7) (Fatty acid-binding protein) (FABP-1) (Glutamate oxaloacetate transaminase 2) (Kynurenine aminotransferase 4) (Kynurenine aminotransferase IV) (Kynurenine--oxoglutarate transaminase 4) (Kynurenine--oxoglutarate transaminase IV) (Plasma membrane-associated fatty acid-binding protein) (FABPpm) (Transaminase A)	61.63	Pathway III
P05977	Myosin light chain 1/3, skeletal muscle isoform (MLC1/MLC3) (MLC1F/MLC3F) (Myosin light chain alkali 1/2) (Myosin light chain A1/A2)	86.17	Pathway III
P08228	Superoxide dismutase [Cu-Zn] (EC 1.15.1.1)	82.47	Pathway III
P08249	Malate dehydrogenase, mitochondrial (EC 1.1.1.37)	61.24	Pathway III
P12787	Cytochrome c oxidase subunit 5A, mitochondrial (Cytochrome c oxidase polypeptide Va)	59.59	Pathway III
P16125	L-lactate dehydrogenase B chain (LDH-B) (EC 1.1.1.27) (LDH heart subunit) (LDH-H)	36.5	Pathway III
P16330	2',3'-cyclic-nucleotide 3'-phosphodiesterase (CNP) (CNPase) (EC 3.1.4.37)	47.1	Pathway III
P17183	Gamma-enolase (EC 4.2.1.11) (2-phospho-D-glycerate hydro-lyase) (Enolase 2) (Neural enolase)	67.74	Pathway III

	(Neuron-specific enolase) (NSE)		
P17742	Peptidyl-prolyl cis-trans isomerase A (PPIase A) (EC 5.2.1.8) (Cyclophilin A) (Cyclosporin A-binding protein) (Rotamase A) (SP18) [Cleaved into: Peptidyl-prolyl cis-trans isomerase A, N-terminally processed]	59.76	Pathway III
P18760	Cofilin-1 (Cofilin, non-muscle isoform)	59.04	Pathway III
P28663	Beta-soluble NSF attachment protein (SNAP-beta) (Brain protein I47) (N-ethylmaleimide-sensitive factor attachment protein beta)	62.42	Pathway III
P31786	Acyl-CoA-binding protein (ACBP) (Diazepam-binding inhibitor) (DBI) (Endozepine) (EP)	57.47	Pathway III
P35979	60S ribosomal protein L12	17.8	Pathway III
P48962	ADP/ATP translocase 1 (ADP,ATP carrier protein 1) (ADP,ATP carrier protein, heart/skeletal muscle isoform T1) (Adenine nucleotide translocator 1) (ANT 1) (Solute carrier family 25 member 4) (mANC1)	55.37	Pathway III
P50396	Rab GDP dissociation inhibitor alpha (Rab GDI alpha) (Guanosine diphosphate dissociation inhibitor 1) (GDI-1)	80.31	Pathway III
P52480	Pyruvate kinase PKM (EC 2.7.1.40) (Pyruvate kinase muscle isozyme)	57.8	Pathway III
P56480	ATP synthase subunit beta, mitochondrial (EC 3.6.3.14)	56.3	Pathway III
P60879	Synaptosomal-associated protein 25 (SNAP-25) (Super protein) (SUP) (Synaptosomal-associated 25 kDa protein)	23.3	Pathway III
P61922	4-aminobutyrate aminotransferase, mitochondrial (EC 2.6.1.19) ((S)-3-amino-2-methylpropionate transaminase) (EC 2.6.1.22) (GABA aminotransferase) (GABA-AT) (Gamma-amino-N-butyrate transaminase) (GABA transaminase) (GABA-T) (L-AIBAT)	56.4	Pathway III
P61982	14-3-3 protein gamma [Cleaved into: 14-3-3 protein gamma, N-terminally processed]	28.3	Pathway III
P62204	Calmodulin (CaM)	76.51	Pathway III
P62259	14-3-3 protein epsilon (14-3-3E)	29.2	Pathway III
P62761	Visinin-like protein 1 (VILIP) (Neural visinin-like protein 1) (NVL-1) (NVP-1)	71.2	Pathway III
P62874	Guanine nucleotide-binding protein G(I)/G(S)/G(T) subunit beta-1 (Transducin beta chain 1)	60.29	Pathway III
P63011	Ras-related protein Rab-3A	55.45	Pathway III
P63038	60 kDa heat shock protein, mitochondrial (60 kDa chaperonin) (Chaperonin 60) (CPN60) (HSP-65) (Heat shock protein 60) (HSP-60) (Hsp60) (Mitochondrial matrix protein P1)	60.9	Pathway III
P63101	14-3-3 protein zeta/delta (Protein kinase C inhibitor protein 1) (KCIP-1) (SEZ-2)	56.33	Pathway III
P63216	Guanine nucleotide-binding protein G(I)/G(S)/G(O) subunit gamma-3	60	Pathway III
P68254	14-3-3 protein theta (14-3-3 protein tau)	27.8	Pathway III

P68368	Tubulin alpha-4A chain (Alpha-tubulin 4) (Alpha-tubulin isotype M-alpha-4) (Tubulin alpha-4 chain)	60.04	Pathway III
P68369	Tubulin alpha-1A chain (Alpha-tubulin 1) (Alpha-tubulin isotype M-alpha-1) (Tubulin alpha-1 chain)	59.42	Pathway III
P68372	Tubulin beta-4B chain (Tubulin beta-2C chain)	71.46	Pathway III
P68510	14-3-3 protein eta	28.2	Pathway III
P70296	Phosphatidylethanolamine-binding protein 1 (PEBP-1) (HCNPpp) [Cleaved into: Hippocampal cholinergic neurostimulating peptide (HCNP)]	76.47	Pathway III
P70349	Histidine triad nucleotide-binding protein 1 (EC 3.-.-.-) (Adenosine 5'-monophosphoramidase) (Protein kinase C inhibitor 1) (Protein kinase C-interacting protein 1) (PKCI-1)	71.43	Pathway III
P97427	Dihydropyrimidinase-related protein 1 (DRP-1) (Collapsin response mediator protein 1) (CRMP-1) (Unc-33-like phosphoprotein 3) (ULIP-3)	58.57	Pathway III
P97457	Myosin regulatory light chain 2, skeletal muscle isoform (Fast skeletal myosin light chain 2) (MLC2F)	66.27	Pathway III
P97870	C-H-ras (Fragment)	89.69	Pathway II+III
P99024	Tubulin beta-5 chain	71.62	Pathway III
P99027	60S acidic ribosomal protein P2	73.04	Pathway III
P99029-2	Peroxiredoxin-5, mitochondrial (EC 1.11.1.15) (Antioxidant enzyme B166) (AOEB166) (Liver tissue 2D-page spot 2D-0014IV) (PLP) (Peroxiredoxin V) (Prx-V) (Peroxisomal antioxidant enzyme) (Thioredoxin peroxidase PMP20) (Thioredoxin reductase)	17	Pathway III
Q05816	Fatty acid-binding protein, epidermal (Epidermal-type fatty acid-binding protein) (E-FABP) (Fatty acid-binding protein 5) (Keratinocyte lipid-binding protein) (Psoriasis-associated fatty acid-binding protein homolog) (PA-FABP)	72.59	Pathway III
Q3U0C4	Putative uncharacterized protein	56.83	Pathway III
Q3U9A8	SH3 domain-binding glutamic acid-rich-like protein	58.77	Pathway III
Q3UDR2	Protein disulfide-isomerase (EC 5.3.4.1) (Fragment)	56.6	Pathway III
Q3UWP8	Putative uncharacterized protein (Fragment)	42.2	Pathway III
Q60932-2	Voltage-dependent anion-selective channel protein 1 (VDAC-1) (mVDAC1) (Outer mitochondrial membrane protein porin 1) (Plasmalemmal porin) (Voltage-dependent anion-selective channel protein 5) (VDAC-5) (mVDAC5)	65.72	Pathway III
Q61171	Peroxiredoxin-2 (EC 1.11.1.15) (Thiol-specific antioxidant protein) (TSA) (Thioredoxin peroxidase 1) (Thioredoxin-dependent peroxide reductase 1)	63.64	Pathway III
Q61598-2	Rab GDP dissociation inhibitor beta (Rab GDI beta) (GDI-3) (Guanosine diphosphate dissociation inhibitor 2) (GDI-2)	46.6	Pathway III

Q63810-2	Calcineurin subunit B type 1 (Protein phosphatase 2B regulatory subunit 1) (Protein phosphatase 3 regulatory subunit B alpha isoform 1)	70	Pathway III
Q68FD5	Clathrin heavy chain 1	191.4	Pathway III
Q6GT24	Peroxiredoxin 6 (Peroxiredoxin-6)	73.21	Pathway III
Q8BGZ1	Hippocalcin-like protein 4 (Neural visinin-like protein 2) (NVP-2)	63.87	Pathway II+III
Q8BWT1	3-ketoacyl-CoA thiolase, mitochondrial (EC 2.3.1.16) (Acetyl-CoA acyltransferase) (Beta-ketothiolase) (Mitochondrial 3-oxoacyl-CoA thiolase)	54.41	Pathway III
Q91V41	Ras-related protein Rab-14	55.35	Pathway III
Q91VB8	Alpha globin 1 (Alpha globin 2) (Alpha-globin) (Hemoglobin alpha, adult chain 1) (Hemoglobin subunit alpha) (Protein Hba-a2)	83.8	Pathway III
Q91XV3	Brain acid soluble protein 1 (22 kDa neuronal tissue-enriched acidic protein) (Neuronal axonal membrane protein NAP-22)	71.68	Pathway III
Q9CQV8-2	14-3-3 protein beta/alpha (Protein kinase C inhibitor protein 1) (KCIP-1) [Cleaved into: 14-3-3 protein beta/alpha, N-terminally processed]	27.8	Pathway III
Q9CQZ1	Heat shock factor-binding protein 1	57.89	Pathway III
Q9D0M5	Dynein light chain 2, cytoplasmic (8 kDa dynein light chain b) (DLC8) (DLC8b) (Dynein light chain LC8-type 2)	57.3	Pathway III
Q9D1G1	Ras-related protein Rab-1B	22.2	Pathway III
Q9D1K2	V-type proton ATPase subunit F (V-ATPase subunit F) (V-ATPase 14 kDa subunit) (Vacuolar proton pump subunit F)	75.63	Pathway III
Q9D6F9	Tubulin beta-4A chain (Tubulin beta-4 chain)	74.32	Pathway III
Q9DB20	ATP synthase subunit O, mitochondrial (Oligomycin sensitivity conferral protein) (OSCP)	55.4	Pathway III
Q9DBJ1	Phosphoglycerate mutase 1 (EC 3.1.3.13) (EC 5.4.2.11) (EC 5.4.2.4) (BPG-dependent PGAM 1) (Phosphoglycerate mutase isozyme B) (PGAM-B)	73.23	Pathway III
A0AUV1	Histone H2A (Fragment)	13.8	Pathway II
A2AFI4	RNA-binding motif protein, X chromosome (Fragment)	54.37	Pathway II+III
A2APL8	Solute carrier family 1 (Glial high affinity glutamate transporter), member 2 (Solute carrier family 1 (Glial high affinity glutamate transporter), member 2, isoform CRA_a)	61.7	Pathway II
A2AQ07	Tubulin beta-1 chain	50.4	Pathway II
A3KGU7	Spectrin alpha chain, non-erythrocytic 1	285	Pathway II+III
B2RWX0	Myosin, heavy polypeptide 1, skeletal muscle, adult	223.2	Pathway II

D3YYN7	Sodium/potassium-transporting ATPase subunit alpha-2	103.5	Pathway II
D3Z041	Long-chain-fatty-acid--CoA ligase 1	78	Pathway II
D3Z7D5	Collagen alpha-2(VI) chain	98.9	Pathway II
E0CZ27	Histone H3 (Fragment)	13.3	Pathway II
E0CZ78	Serine/threonine-protein phosphatase (EC 3.1.3.16)	59	Pathway II
E9PV73	Neuron-specific calcium-binding protein hippocalcin (Fragment)	21.8	Pathway II
E9PZF0	Nucleoside diphosphate kinase (EC 2.7.4.6)	30.2	Pathway II+III
E9Q1V9	Calcium/calmodulin-dependent protein kinase type II subunit delta	57.7	Pathway II
F8WJK8	Hsc70-interacting protein	40.5	Pathway II
G3UW82	MCG140437, isoform CRA_d (Protein Myh2)	223.1	Pathway II
G3UX26	Voltage-dependent anion-selective channel protein 2 (Fragment)	30.4	Pathway II
G3XA10	Heterogeneous nuclear ribonucleoprotein U (Heterogeneous nuclear ribonucleoprotein U, isoform CRA_b)	86.8	Pathway II
O08749	Dihydrolipoyl dehydrogenase, mitochondrial (EC 1.8.1.4) (Dihydrolipoamide dehydrogenase)	54.2	Pathway II
O35129	Prohibitin-2 (B-cell receptor-associated protein BAP37) (Repressor of estrogen receptor activity)	33.3	Pathway II
O35737	Heterogeneous nuclear ribonucleoprotein H (hnRNP H) [Cleaved into: Heterogeneous nuclear ribonucleoprotein H, N-terminally processed]	49.2	Pathway II
O55125	Protein NipSnap homolog 1 (NipSnap1)	33.3	Pathway II
O70251	Elongation factor 1-beta (EF-1-beta)	24.7	Pathway II
P05063	Fructose-bisphosphate aldolase C (EC 4.1.2.13) (Aldolase 3) (Brain-type aldolase) (Scrapie-responsive protein 2) (Zebrin II)	39.4	Pathway II
P07724	Serum albumin	68.6	Pathway II+III
P09411	Phosphoglycerate kinase 1 (EC 2.7.2.3)	44.5	Pathway II+III
P10637	Microtubule-associated protein tau (Neurofibrillary tangle protein) (Paired helical filament-tau) (PHF-tau)	46.5	Pathway II
P11499	Heat shock protein HSP 90-beta (Heat shock 84 kDa) (HSP 84) (HSP84) (Tumor-specific transplantation 84 kDa antigen) (TSTA)	83.2	Pathway II+III
P11798	Calcium/calmodulin-dependent protein kinase type II subunit alpha (CaM kinase II subunit alpha) (CaMK-II subunit alpha) (EC 2.7.11.17)	54.1	Pathway II
P13542	Myosin-8 (Myosin heavy chain 8) (Myosin heavy chain, skeletal muscle, perinatal) (MyHC-perinatal)	222.6	Pathway II
P13595	Neural cell adhesion molecule 1 (N-CAM-1) (NCAM-1) (CD antigen CD56)	119.4	Pathway II

P14148	60S ribosomal protein L7	31.4	Pathway II
P15864	Histone H1.2 (H1 VAR.1) (H1c)	21.3	Pathway II
P16858	Glyceraldehyde-3-phosphate dehydrogenase (GAPDH) (EC 1.2.1.12) (Peptidyl-cysteine S-nitrosylase GAPDH) (EC 2.6.99.-)	35.8	Pathway II+III
P17182	Alpha-enolase (EC 4.2.1.11) (2-phospho-D-glycerate hydro-lyase) (Enolase 1) (Non-neural enolase) (NNE)	70.74	Pathway II+III
P17426	AP-2 complex subunit alpha-1 (100 kDa coated vesicle protein A) (Adaptor protein complex AP-2 subunit alpha-1) (Adaptor-related protein complex 2 subunit alpha-1) (Alpha-adaptin A) (Alpha1-adaptin) (Clathrin assembly protein complex 2 alpha-A large chain) (Plasma membrane adaptor HA2/AP2 adaptin alpha A subunit)	107.6	Pathway II
P19253	60S ribosomal protein L13a (Transplantation antigen P198) (Tum-P198 antigen)	23.4	Pathway II
P19536	Cytochrome c oxidase subunit 5B, mitochondrial (Cytochrome c oxidase polypeptide Vb)	13.8	Pathway II
P26041	Moesin (Membrane-organizing extension spike protein)	67.7	Pathway II
P26043	Radixin (ESP10)	68.5	Pathway II
P26443	Glutamate dehydrogenase 1, mitochondrial (GDH 1)	61.3	Pathway II
P35700	Peroxiredoxin-1 (EC 1.11.1.15) (Macrophage 23 kDa stress protein) (Osteoblast-specific factor 3) (OSF-3) (Thioredoxin peroxidase 2) (Thioredoxin-dependent peroxide reductase 2)	72.86	Pathway II+III
P39053-3	Dynamin-1 (EC 3.6.5.5)	95.9	Pathway II
P40142	Transketolase (TK) (EC 2.2.1.1) (P68)	67.6	Pathway II
P41105	60S ribosomal protein L28	15.7	Pathway II
P41216	Long-chain-fatty-acid--CoA ligase 1	77.9	Pathway II
P42932	T-complex protein 1 subunit theta (TCP-1-theta) (CCT-theta)	59.5	Pathway II
P43006	Excitatory amino acid transporter 2 (GLT-1) (Sodium-dependent glutamate/aspartate transporter 2) (Solute carrier family 1 member 2)	62	Pathway II
P46460	Vesicle-fusing ATPase)	82.6	Pathway II
P47738	Aldehyde dehydrogenase, mitochondrial (EC 1.2.1.3) (AHD-M1) (ALDH class 2) (ALDH-E2) (ALDHI)	56.5	Pathway II
P48453	Serine/threonine-protein phosphatase 2B catalytic subunit beta isoform (EC 3.1.3.16) (CAM-PRP catalytic subunit) (Calmodulin-dependent calcineurin A subunit beta isoform)	59.1	Pathway II
P50247	Adenosylhomocysteinase (AdoHcyase) (S-adenosyl-L-homocysteine hydrolase)	47.7	Pathway II
P50516	V-type proton ATPase catalytic subunit A (V-ATPase subunit A)	68.3	Pathway II+III
P53810	Phosphatidylinositol transfer protein alpha isoform (PI-TP-alpha) (PtdIns transfer protein alpha)	31.9	Pathway II

	(PtdInsTP alpha)		
P56375	Acylphosphatase-2 (EC 3.6.1.7) (Acylphosphatase, muscle type isozyme) (Acylphosphate phosphohydrolase 2)	11.9	Pathway II
P57780	Alpha-actinin-4 (F-actin cross-linking protein) (Non-muscle alpha-actinin 4)	104.9	Pathway II
P60710	Actin, cytoplasmic 1 (Beta-actin) [Cleaved into: Actin, cytoplasmic 1, N-terminally processed]	31.5	Pathway II
P61089	Ubiquitin-conjugating enzyme E2 N	17.1	Pathway II
P61205	ADP-ribosylation factor 3	58.56	Pathway II+III
P61226	Ras-related protein Rap-2b	20.5	Pathway II
P63017	Heat shock cognate 71 kDa protein (Heat shock 70 kDa protein 8)	70.8	Pathway II+III
P63054	Purkinje cell protein 4 (Brain-specific antigen PCP-4) (Brain-specific polypeptide PEP-19)	6.8	Pathway II
P63325	40S ribosomal protein S10	18.9	Pathway II
P63328	Serine/threonine-protein phosphatase 2B catalytic subunit alpha isoform (CAM-PRP catalytic subunit) (Calmodulin-dependent calcineurin A subunit alpha isoform)	58.6	Pathway II
P68040	Guanine nucleotide-binding protein subunit beta-2-like 1 (12-3) (Receptor for activated C kinase) (Receptor of activated protein kinase C 1) (RACK1) (p205) [Cleaved into: Guanine nucleotide-binding protein subunit beta-2-like 1, N-terminally processed]	35.1	Pathway II+III
P68134	Actin, alpha skeletal muscle (Alpha-actin-1)	69.76	Pathway II+III
P80318	T-complex protein 1 subunit gamma (TCP-1-gamma) (CCT-gamma) (Matricin) (mTRiC-P5)	60.6	Pathway II
P84084	ADP-ribosylation factor 5	20.5	Pathway II
Q02053	Ubiquitin-like modifier-activating enzyme 1 (Ubiquitin-activating enzyme E1) (Ubiquitin-activating enzyme E1 X) (Ubiquitin-like modifier-activating enzyme 1 X)	117.7	Pathway II
Q03265	ATP synthase subunit alpha, mitochondrial	59.7	Pathway II
Q04447	Creatine kinase B-type	42.7	Pathway II+III
Q05DU4	Msn protein (Fragment)	51.9	Pathway II
Q09J72	Myelin basic protein (Fragment)	15.6	Pathway II
Q3TFE8	Putative uncharacterized protein	97.1	Pathway II
Q3THQ5	Putative uncharacterized protein	62.5	Pathway II
Q3THU8	Putative uncharacterized protein	39.6	Pathway II
Q3TKD0	Transportin-1 (Fragment)	91.7	Pathway II
Q3U8D2	Putative uncharacterized protein	41.5	Pathway II

Q3UF82	Putative uncharacterized protein	40.6	Pathway II
Q3ULJ0-2	Glycerol-3-phosphate dehydrogenase 1-like protein	34.6	Pathway II
Q547J4	NADH dehydrogenase subunit 5 (Fragment)	27.5	Pathway II
Q5D0A4	Stx1a protein (Fragment)	59.93	Pathway II+III
Q5EBQ0	Voltage-dependent anion channel 3	30.9	Pathway II
Q60930	Voltage-dependent anion-selective channel protein 2 (VDAC-2) (mVDAC2) (Outer mitochondrial membrane protein porin 2) (Voltage-dependent anion-selective channel protein 6) (VDAC-6) (mVDAC6)	31.7	Pathway II
Q61885	Myelin-oligodendrocyte glycoprotein	28.3	Pathway II
Q64521	Glycerol-3-phosphate dehydrogenase, mitochondrial (GPD-M) (GPDH-M)	80.9	Pathway II
Q684I8	Isocitrate dehydrogenase 3 (NAD+), gamma (Fragment)	38.3	Pathway II
Q6PIC6	Sodium/potassium-transporting ATPase subunit alpha-3	111.6	Pathway II
Q6PIE5	Sodium/potassium-transporting ATPase subunit alpha-2	112.1	Pathway II
Q6ZWS7	Putative uncharacterized protein	55.9	Pathway II
Q71LX8	Heat shock protein 84b (Heat shock protein 90 alpha (Cytosolic), class B member 1) (MCG18238)	253.5	Pathway II
Q7TMM9	Tubulin beta-2A chain	71.46	Pathway II+III
Q80SW1	Putative adenosylhomocysteinase 2 (AdoHcyase 2)	58.9	Pathway II
Q80XN0	D-beta-hydroxybutyrate dehydrogenase, mitochondrial (BDH) (EC 1.1.1.30) (3-hydroxybutyrate dehydrogenase)	38.3	Pathway II
Q8BIZ1	Ankyrin repeat and sterile alpha motif domain-containing protein 1B	139	Pathway II
Q8BMS1	Trifunctional enzyme subunit alpha, mitochondrial (TP-alpha)	82.6	Pathway II
Q8BUT2	Putative uncharacterized protein	92.8	Pathway II
Q8BZM2	Putative uncharacterized protein (Fragment)	52.3	Pathway II
Q8C0M9	Isoaspartyl peptidase/L-asparaginase	33.9	Pathway II
Q8C1B7	Septin-11	49.7	Pathway II
Q8C553	Putative uncharacterized protein (Fragment)	47.5	Pathway II
Q8C8R3	Ankyrin-2 (ANK-2) (Brain ankyrin)	52.8	Pathway II
Q8CAQ8	Serine protease inhibitor Kazal-type 10	83.8	Pathway II
Q8R5H6	Wiskott-Aldrich syndrome protein family member 1 (WASP family protein member 1) (Protein WAVE-	61.5	Pathway II

	1)		
Q8VEH3	ADP-ribosylation factor-like protein 8A (ADP-ribosylation factor-like protein 10B) (Novel small G protein indispensable for equal chromosome segregation 2)	21.3	Pathway II
Q91V12	Cytosolic acyl coenzyme A thioester hydrolase	42.5	Pathway II
Q920I9	WD repeat-containing protein 7 (TGF-beta resistance-associated protein TRAG)	163.3	Pathway II
Q9CPU0	Lactoylglutathione lyase	20.8	Pathway II
Q9CR62	Mitochondrial 2-oxoglutarate/malate carrier protein (OGCP) (Solute carrier family 25 member 11)	34.1	Pathway II
Q9CWJ9	Bifunctional purine biosynthesis protein PURH	64.2	Pathway II
Q9D023	Mitochondrial pyruvate carrier 2 (Brain protein 44)	14.3	Pathway II
Q9D819	Inorganic pyrophosphatase (EC 3.6.1.1) (Pyrophosphate phospho-hydrolase) (PPase)	32.6	Pathway II
Q9DBG3	AP-2 complex subunit beta (AP105B) (Adaptor protein complex AP-2 subunit beta) (Adaptor-related protein complex 2 subunit beta) (Beta-2-adaptin) (Beta-adaptin) (Clathrin assembly protein complex 2 beta large chain) (Plasma membrane adaptor HA2/AP2 adaptin beta subunit)	104.5	Pathway II
Q9DCX2	ATP synthase subunit d, mitochondrial (ATPase subunit d)	18.7	Pathway II
Q9ERD7	Tubulin beta-3 chain	50.4	Pathway II
Q9ERE2	Keratin, type II cuticular Hb1 (Keratin-81) (K81) (Type II hair keratin Hb1)	43.7	Pathway II
Q9QYR6	Microtubule-associated protein 1A (MAP-1A) [Cleaved into: MAP1A heavy chain; MAP1 light chain LC2]	300	Pathway II
Q9Z1Z0	General vesicular transport factor p115 (Protein USO1 homolog) (Transcytosis-associated protein) (TAP) (Vesicle-docking protein)	106.9	Pathway II

Table S5 shows the identified proteins with accession number, protein name, protein mass, predominant structure and from which pathway from the workflow it comes from. It also shows the number of images of tryptic peptides which shows a structure (every structure, not only the predominant structure) corresponding to histology (**# images with structure)**. Furthermore it displays the number of finally identified tryptic peptide sequences (**# Peptide sequences for predominant structure)** and how many additional adducts could be found. Column 6 shows the number of unique peptides of these peptide sequences in comparison with all tryptic peptides found in our workflow.

Table S5 Identified proteins with their tryptic peptides for coronal mouse brain section measured with 50 µm pixel size

**Accession no**	**Protein**	**mass (kDa)**	**# images with structure**	**# Peptide sequences with predominant structure**	**# unique peptides**	**# adducts**	**structure**	**protein origin**
A0AUV1	Histone H2A (Fragment)	13.8	10	6	5	2	*Ependyma*	Pathway II
A3KGU7	Spectrin alpha chain, non-erythrocytic 1	285	54	25	22	4	*Cortex*	Pathway II+III
B2RWX0	Myosin, heavy polypeptide 1, skeletal muscle, adult	223.2	18	8	8	2	*Corpus Callosum*	Pathway II
D3Z7D5	Collagen alpha-2(VI) chain	98.9	21	13	13	2	*Cortex*	Pathway II
E0CZ27	Histone H3 (Fragment)	13.3	7	4	3	0	*Ependyma*	Pathway II
E0CZ78	Serine/threonine-protein phosphatase	59	6	4	1	1	*Cortex*	Pathway II
E9PV73	Neuron-specific calcium-binding protein hippocalcin (Fragment)	21.8	5	3	2	0	*Corpus Callosum*	Pathway II
E9PZF0	Nucleoside diphosphate kinase	30.2	5	3	2	0	*Corpus Callosum*	Pathway II+III
E9Q1V9	Calcium/calmodulin-dependent protein kinase type II subunit delta	57.7	6	4	2	1	*Cortex*	Pathway II
F8WJK8	Hsc70-interacting protein	40.5	15	8	6	2	*Cortex*	Pathway II
G3UW82	MCG140437, isoform CRA_d	223.1	41	21	12	1	*Cortex*	Pathway II
G3XA10	Heterogeneous nuclear ribonucleoprotein U	86.8	13	8	8	0	*Cortex*	Pathway II
O08553	Dihydropyrimidinase-related protein 2	62.2	6	5	4	0	*Ependyma*	Pathway III
O08599	Syntaxin-binding protein 1	67.5	16	7	6	1	*Cortex*	Pathway III
O35129	Prohibitin-2	33.3	5	3	2	0	*Ependyma*	Pathway II
O35737	Heterogeneous nuclear ribonucleoprotein H	49.2	5	2	1	0	*Corpus Callosum*	Pathway II
O55125	Protein NipSnap homolog 1	33.3	7	5	4	0	*Corpus Callosum*	Pathway II

O70251	Elongation factor 1-beta	24.7	5	2	1	0	*Cortex*	Pathway II
P00920	Carbonic anhydrase 2	29	5	3	3	0	*Ependyma*	Pathway III
P05063	Fructose-bisphosphate aldolase C	39.4	4	4	3	0	*Cortex*	Pathway III
P05064	Fructose-bisphosphate aldolase A	39.3	3	2	2	1	*Cortex*	Pathway III
P07724	Serum albumin	68.6	5	3	2	0	*Corpus Callosum*	Pathway II+III
P09411	Phosphoglycerate kinase 1	44.5	8	6	6	1	*Cortex*	Pathway II+III
P10637	Eukaryotic initiation factor 4A-II (Isoform 2)	46.5	6	3	2	2	*Ependyma*	Pathway II
P11499	Heat shock protein HSP 90-beta	83.2	7	4	3	0	*Corpus Callosum*	Pathway II+III
P11798	Calcium/calmodulin-dependent protein kinase type II subunit alpha	54.1	6	4	1	0	*Cortex*	Pathway II
P13542	Myosin-8	222.6	17	9	3	0	*Cortex*	Pathway II
P13595	Neural cell adhesion molecule 1	119.4	6	4	4	0	*Cortex*	Pathway II
P14148	60S ribosomal protein L7	31.4	11	6	6	1	*Cortex*	Pathway II
P15864	Histone H1.2	21.3	15	10	6	4	*Ependyma*	Pathway II
P16125	L-lactate dehydrogenase B chain	36.5	4	2	2	0	*Cortex*	Pathway III
P16330	2',3'-cyclic-nucleotide 3'-phosphodiesterase	47.1	7	3	3	1	*Corpus Callosum*	Pathway III
P16858	Glyceraldehyde-3-phosphate dehydrogenase	35.8	3	3	3	0	*Cortex*	Pathway II+III
P17426	AP-2 complex subunit alpha-1	107.6	13	7	5	2	*Cortex*	Pathway II
P19253	60S ribosomal protein L13a	23.4	5	2	2	0	*Cortex*	Pathway II
P26041	Moesin	67.7	12	5	3	0	*Cortex*	Pathway II
P26443	Glutamate dehydrogenase 1, mitochondrial	61.3	9	4	4	0	*Corpus Callosum*	Pathway II
P35979	60S ribosomal protein L12	17.8	3	2	2	0	*Cortex*	Pathway III
P39053-3	Dynamin-1 (Isoform 3)	95.9	8	6	4	0	*Cortex*	Pathway II
P41105	60S ribosomal protein L28	15.7	4	2	2	0	*Cortex*	Pathway II
P41216	Long-chain-fatty-acid--CoA ligase 1	77.9	7	5	1	0	*Cortex*	Pathway II
P42932	T-complex protein 1 subunit theta	59.5	5	2	2	0	*Corpus Callosum*	Pathway II
P43006	Excitatory amino acid transporter 2	62	5	3	2	0	*Cortex*	Pathway II

P46460	Vesicle-fusing ATPase	82.6	4	3	3	0	*Cortex*	Pathway II
P47738	Aldehyde dehydrogenase, mitochondrial	56.5	7	5	3	0	*Cortex*	Pathway II
P50247	Adenosylhomocysteinase	47.7	3	3	3	0	*Cortex*	Pathway II
P50516	V-type proton ATPase catalytic subunit A	68.3	6	3	3	1	*Corpus Callosum*	Pathway II+III
P52480	Thiosulfate sulfurtransferase	57.8	7	4	3	1	*Cortex*	Pathway III
P53810	Phosphatidylinositol transfer protein alpha isoform	31.9	6	3	3	2	*Corpus Callosum*	Pathway II
P56480	ATP synthase subunit beta, mitochondrial	56.3	5	3	2	1	*Cortex*	Pathway III
P60879	Synaptosomal-associated protein 25	23.3	6	3	2	0	*Corpus Callosum*	Pathway III
P61922	4-aminobutyrate aminotransferase, mitochondrial	56.4	4	3	3	0	*Corpus Callosum*	Pathway III
P61982	14-3-3 protein gamma	28.3	5	3	3	1	*Ependyma*	Pathway III
P62259	14-3-3 protein epsilon	29.2	4	3	3	1	*Ependyma*	Pathway III
P63017	Heat shock cognate 71 kDa protein	70.8	10	6	5	1	*Cortex*	Pathway III
P63038	60 kDa heat shock protein, mitochondrial	60.9	10	5	3	0	*Cortex*	Pathway II+III
P63054	Purkinje cell protein 4	6.8	4	2	2	0	*Corpus Callosum*	Pathway II
P63325	40S ribosomal protein S10	18.9	8	6	6	2	*Corpus Callosum*	Pathway II
P63328	Serine/threonine-protein phosphatase 2B catalytic subunit alpha isoform	58.6	7	4	1	0	*Cortex*	Pathway II
P68040	Guanine nucleotide-binding protein subunit beta-2-like 1	35.1	4	2	1	2	*Cortex*	Pathway II+III
P68254	14-3-3 protein theta	27.8	4	2	2	0	*Cortex*	Pathway III
P68510	14-3-3 protein eta	28.2	5	3	3	0	*Cortex*	Pathway III
Q02053	Ubiquitin-like modifier-activating enzyme 1	117.7	3	2	2	0	*Cortex*	Pathway II
Q03265	ATP synthase subunit alpha, mitochondrial	59.7	6	3	1	0	*Cortex*	Pathway II

Q04447	Creatine kinase B-type	42.7	4	2	1	0	*Corpus Callosum*	Pathway II+III
Q09J72	Myelin basic protein (Fragment)	15.6	21	9	9	9	*Corpus Callosum*	Pathway II
Q3TFE8	Putative uncharacterized protein	97.1	6	4	4	2	*Corpus Callosum*	Pathway II
Q3THQ5	Putative uncharacterized protein	62.5	14	9	6	1	*Corpus Callosum*	Pathway II
Q3THU8	Putative uncharacterized protein	39.6	4	2	2	0	*Corpus Callosum*	Pathway II
Q3TKD0	Transportin-1 (Fragment)	91.7	3	2	2	0	*Cortex*	Pathway II
Q3UDR2	Putative uncharacterized protein (Fragment)	56.6	8	3	3	2	*Corpus Callosum*	Pathway III
Q3UF82	Putative uncharacterized protein	40.6	4	3	2	0	*Ependyma*	Pathway II
Q3ULJ0-2	Glycerol-3-phosphate dehydrogenase 1-like protein (Isoform 2)	34.6	2	2	2	0	*Corpus Callosum*	Pathway II
Q3UWP8	Putative uncharacterized protein (Fragment)	42.2	2	2	2	0	*Cortex*	Pathway III
Q547J4	Putative uncharacterized protein	27.5	8	5	4	1	*Corpus Callosum*	Pathway II
Q61598-2	Rab GDP dissociation inhibitor beta (Isoform 2)	46.6	4	2	2	0	*Cortex*	Pathway III
Q61885	Myelin-oligodendrocyte glycoprotein	28.3	2	2	2	0	*Ependyma*	Pathway II
Q64521	Glycerol-3-phosphate dehydrogenase, mitochondrial	80.9	3	3	2	1	*Cortex*	Pathway II
Q684I8	Isocitrate dehydrogenase 3 (NAD+), gamma (Fragment)	38.3	5	3	2	0	*Cortex*	Pathway II
Q68FD5	Clathrin heavy chain 1	191.4	9	5	4	1	*Ependyma*	Pathway III
Q6PIC6	Sodium/potassium-transporting ATPase subunit alpha-3	111.6	7	4	2	0	*Corpus Callosum*	Pathway II
Q6PIE5	Sodium/potassium-transporting ATPase subunit alpha-2	112.1	9	5	2	0	*Cortex*	Pathway II
Q71LX8	Talin-2	253.5	3	2	2	0	*Ependyma*	Pathway II
Q80SW1	Putative adenosylhomocysteinase 2	58.9	8	3	3	1	*Corpus Callosum*	Pathway II

Q8BIZ1	Ankyrin repeat and sterile alpha motif domain-containing protein 1B	139	9	6	6	1	*Cortex*	Pathway II
Q8BMS1	Trifunctional enzyme subunit alpha, mitochondrial	82.6	10	5	4	0	*Corpus Callosum*	Pathway II
Q8C0M9	Isoaspartyl peptidase/L-asparaginase	33.9	5	2	1	0	*Cortex*	Pathway II
Q8C1B7	Septin-11	49.7	8	6	6	2	*Cortex*	Pathway II
Q8C8R3	Putative uncharacterized protein	52.8	26	13	11	2	*Cortex*	Pathway II
Q8R5H6	Wiskott-Aldrich syndrome protein family member 1	61.5	6	3	2	0	*Cortex*	Pathway II
Q91V12	Cytosolic acyl coenzyme A thioester hydrolase	42.5	7	3	3	1	*Cortex*	Pathway II
Q920I9	WD repeat-containing protein 7	163.3	5	2	2	0	*Corpus Callosum*	Pathway II
Q9CPU0	Lactoylglutathione lyase	20.8	2	2	2	0	*Corpus Callosum*	Pathway II
Q9CR62	Mitochondrial 2-oxoglutarate/malate carrier protein	34.1	3	2	2	0	*Cortex*	Pathway II
Q9D023	Mitochondrial pyruvate carrier 2	14.3	5	2	2	0	*Corpus Callosum*	Pathway II
Q9D1G1	Ras-related protein Rab-1B	22.2	4	3	2	0	*Corpus Callosum*	Pathway III
Q9DBG3	AP-2 complex subunit beta	104.5	6	2	2	1	*Cortex*	Pathway II
Q9ERD7	Tubulin beta-3 chain	50.4	4	2	1	1	*Ependyma*	Pathway II+III
Q9Z1Z0	General vesicular transport factor p115	106.9	5	3	3	1	*Cortex*	Pathway II
**In total**			**772**	**434**	**342**	**67**		

III. MALDI MS imaging of a coronal mouse brain section with 25 µm pixel size

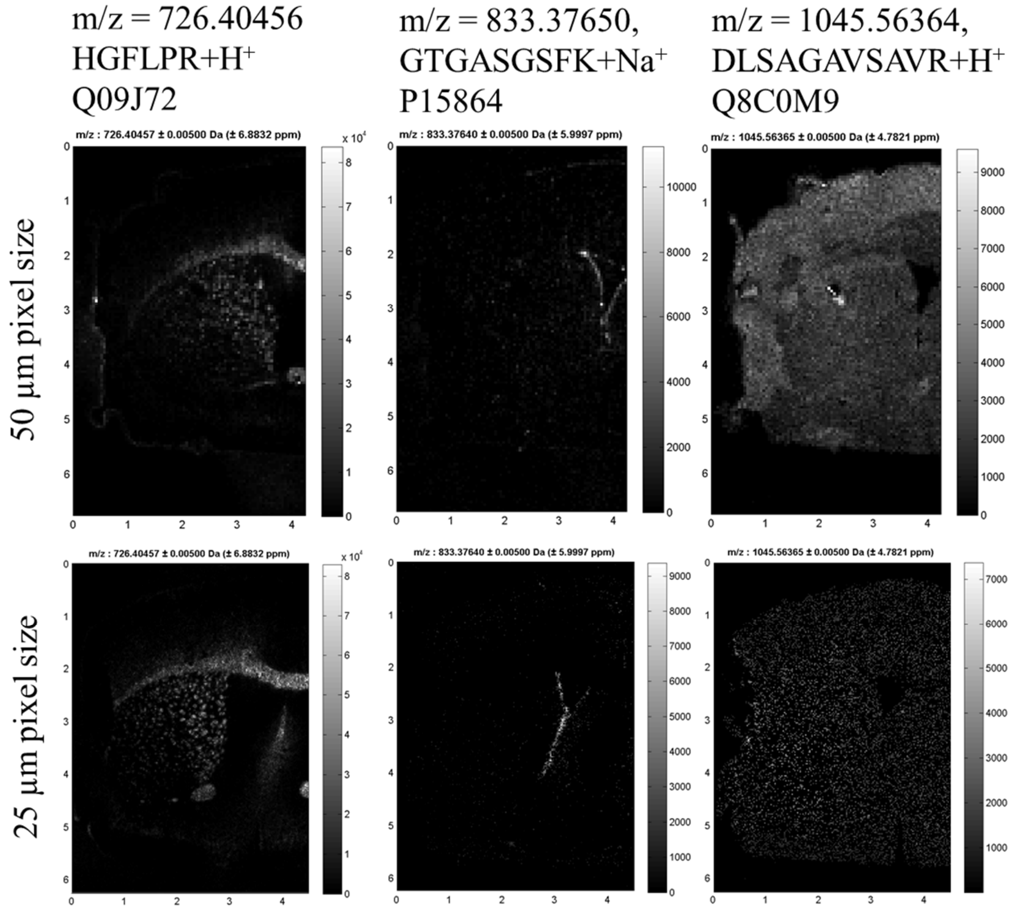

Fig. S5 Comparison of 25 µm und 50 µm pixel size. Measurements correspond to Figure 1 and Figure 3, respectively

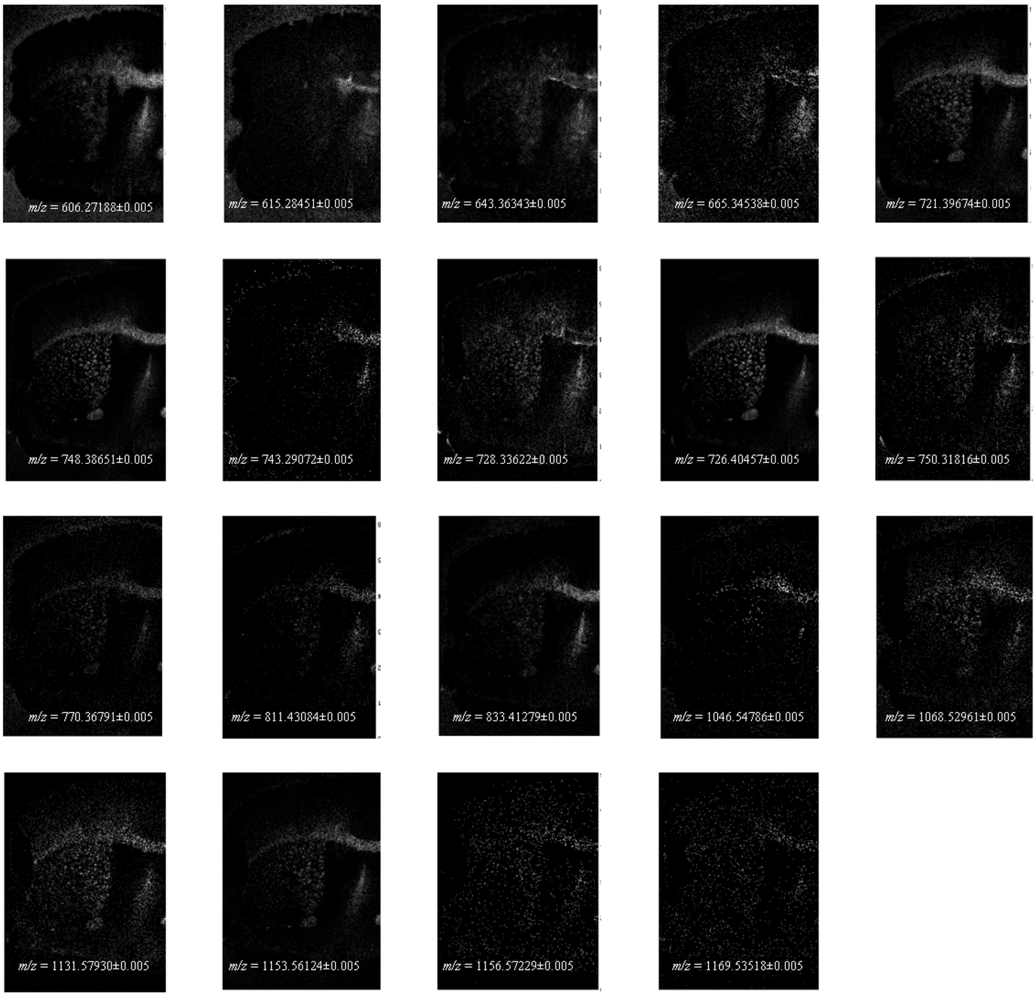

Fig. S6 Tryptic peptides of myelin basic protein with 25 μm pixel size

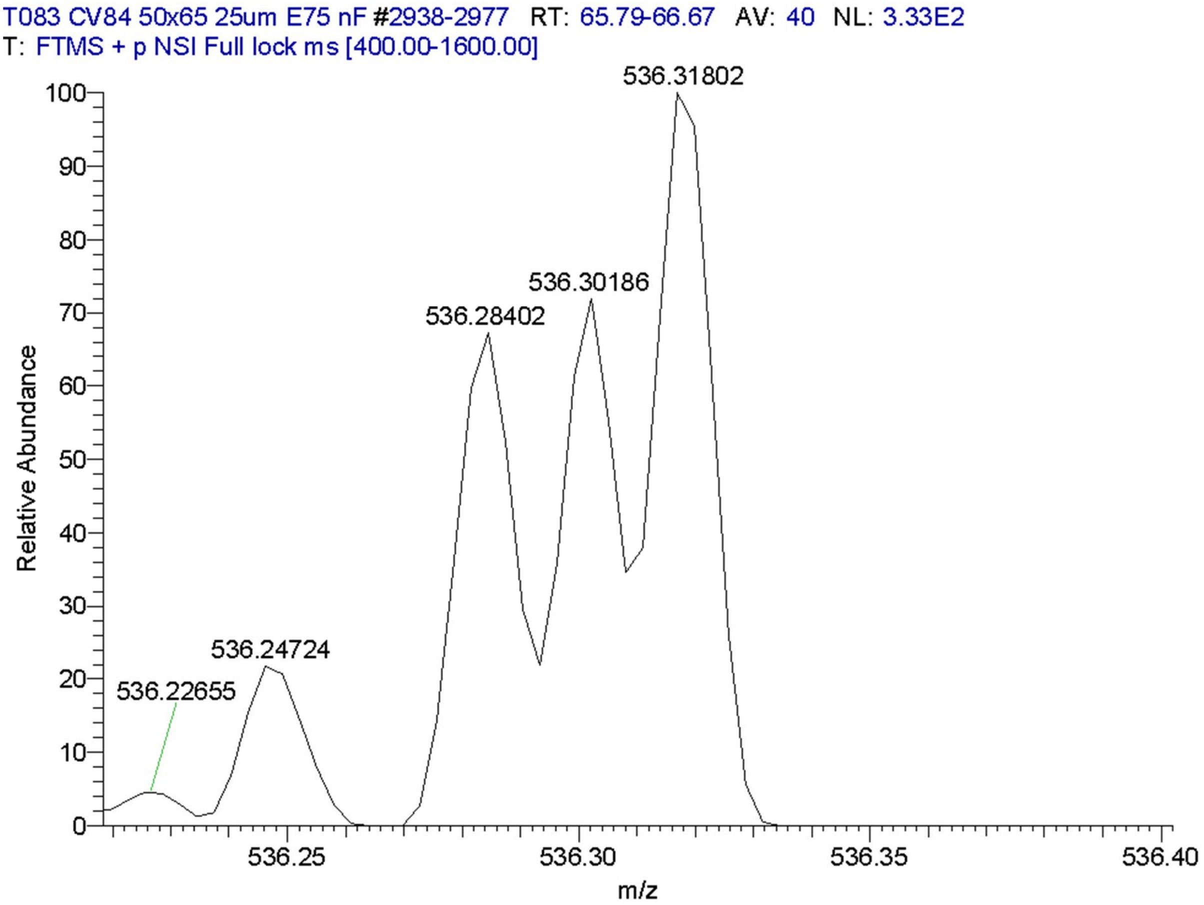

Fig. S7 Average spectrum of the peptide peak m/z = 536.3173 and neighboring peaks

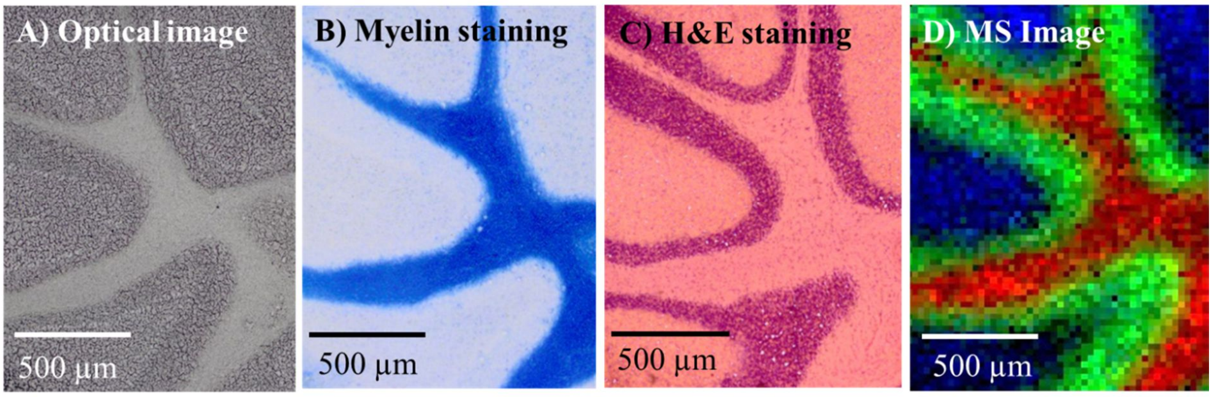

Fig. S8 MS imaging of coronal mouse brain cerebellum with a pixel size of 25 µm A) Optical image B) Myelin staining C) H&E staining D) RGB image of tryptic peptides: m/z = 726.4045± 0.005,, HGFLPR + H^+ (red) m/z = 536.3173 ± 0.005, AKPAK + Na^+ (green) and m/z = 994.5436 ± 0.005, WRQLIEK + Na^+ (blue)
